# An International Commentary on Dysphagia and Dysphonia During the COVID-19 Pandemic

**DOI:** 10.1007/s00455-021-10396-z

**Published:** 2022-01-04

**Authors:** Anna Miles, Jackie McRae, Gemma Clunie, Patricia Gillivan-Murphy, Yoko Inamoto, Hanneke Kalf, Mershen Pillay, Susan Pownall, Philippa Ratcliffe, Theresa Richard, Ursula Robinson, Sarah Wallace, Martin B. Brodsky

**Affiliations:** 1 Speech Science, School of Psychology, The University of Auckland, Grafton Campus, Private Bag 92019, Auckland, New Zealand; 2 Centre for Allied Health, St George’s, University of London/University College London Hospitals NHS Foundation Trust, London, UK; 3 Imperial College London & Clinical Specialist SLT (Airways/ENT), Imperial College Healthcare NHS Trust, London, UK; 4 Clinical Specialist SLT, Voice & Swallowing Clinic, Mater Misericordiae University Hospital, Dublin, Ireland; 5 SLHT, Faculty of Rehabilitation, School of Health Sciences, Fujita Health University, Toyoake, Japan; 6 Division of Speech Pathology, Department of Rehabilitation, Radboud University Medical Centre / Donders Centre for Neuroscience, Nijmegen, The Netherlands; 7 Speech-Language Therapy, University of KwaZulu-Natal, Durban, South Africa; 8 Sheffield Teaching Hospitals NHS Foundation Trust, Sheffield, UK; 9 Consultant SLT Royal National ENT and EDH University College London Hospitals NHS Foundation Trust, London, UK; 10 Mobile Dysphagia Diagnostics, Medical SLP Collective, Buffalo, USA; 11 SLT, Belfast City Hospital, Belfast Health & Social Care Trust, Belfast, UK; 12 Consultant SLT, Wythenshawe Hospital, Manchester University NHS Foundation Trust, Manchester, UK; 13 Division of Pulmonary and Critical Care Medicine, Department of Physical Medicine and Rehabilitation, Outcomes After Critical Illness and Surgery (OACIS) Research Group, Johns Hopkins University, Baltimore, MD USA

**Keywords:** COVID-19, Dysphagia, Deglutition, Dysphonia, ICU, Voice

## Abstract

COVID-19 has had an impact globally with millions infected, high mortality, significant economic ramifications, travel restrictions, national lockdowns, overloaded healthcare systems, effects on healthcare workers’ health and well-being, and large amounts of funding diverted into rapid vaccine development and implementation. Patients with COVID-19, especially those who become severely ill, have frequently developed dysphagia and dysphonia. Health professionals working in the field have needed to learn about this new disease while managing these patients with enhanced personal protective equipment. Emerging research suggests differences in the clinical symptoms and journey to recovery for patients with COVID-19 in comparison to other intensive care populations. New insights from outpatient clinics also suggest distinct presentations of dysphagia and dysphonia in people after COVID-19 who were not hospitalized or severely ill. This international expert panel provides commentary on the impact of the pandemic on speech pathologists and our current understanding of dysphagia and dysphonia in patients with COVID-19, from acute illness to long-term recovery. This narrative review provides a unique, comprehensive critical appraisal of published peer-reviewed primary data as well as emerging previously unpublished, original primary data from across the globe, including clinical symptoms, trajectory, and prognosis. We conclude with our international expert opinion on what we have learnt and where we need to go next as this pandemic continues across the globe.

## Introduction

Coronavirus disease 2019 (COVID-19) is a viral disease caused by the highly infectious respiratory pathogen severe acute respiratory syndrome coronavirus 2 (SARS-CoV-2). Since its discovery in China in December 2019, COVID-19 has spread globally, being declared a pandemic by the World Health Organization on March 11, 2020. The first six months of the pandemic led to a rapid response from otolaryngology (ENT) and speech pathology associations to provide guidance on infection control practices, with a focus on physical distancing, equipment disinfection, adaptations to practice, and personal protective equipment (PPE), particularly for potential aerosol generating procedures and behaviors (AGPs) [1–3].

In this paper, the international expert panel provides commentary on the impact of the pandemic on health professionals working with patients with dysphagia and our current understanding of dysphagia and dysphonia in patients with COVID-19, from acute illness to long-term recovery. This narrative review provides a comprehensive, critical appraisal of 15 published studies with a combined 1112 patients focused on dysphagia and dysphonia after COVID-19. We also provided preliminary insights from emerging, unpublished primary data from 9 international sites (698 patients). These unpublished sets of data each have ethics committee approvals. Additional details are presented in Table 20 and in the acknowledgements. Finally, we also report on survey data from 2353 speech pathologists (SPs), physicians, and dentists, including data previously only available in Japanese. We conclude with lessons learned and next steps as this pandemic continues across the globe.

## Effect on Clinical Practice

Surveys have been conducted worldwide, some focused on infection control and restrictions in caseload management and some focused on the uptake and attitudes toward telehealth, while others focused on health professionals’ well-being. We describe a selection of surveys from across the world in order to explore the impact of the pandemic on those working in dysphagia care. In an early survey by the Royal College of Speech and Language Therapists (RCSLT) of 544 UK SPs in April 2020 [4] “96% of respondents said that the pandemic was having an impact on their professional roles, responsibilities, and duties.” Of concern, 49% of SPs were not seeing patients and 63% reported a reduction in their routine clinical caseloads. One study compared referrals to SP services in 2020 to the same period in 2019 and found substantially less referrals across all services for neurorehabilitation [5]. Twenty percent of SPs reported redeployment in April/May 2020 and by August/September 2020; 76% of SP service managers reported a lower workforce capacity associated with redeployment [4, 6]. This follow-up RCSLT survey in August /September 2020 with 413 SP respondents found that the reduced communication referrals slowed from 38 to 24%, but there was a 7% increase in dysphagia referrals [6]. These global reductions in routine caseload numbers were likely multifactorial, including time-consuming infection control practices, workplace closures, patients declining due to personal infection risk concerns, prohibition of AGPs, and clinical activity being paused to redeploy the workforce to COVID-19 and critical care areas. Referrals from general practitioners and otolaryngology, for example, were significantly reduced in the UK. Similarly, routine referrals from aged-care facilities were reduced to limit the spread of infection.

In Ireland, there were similar trends. Two surveys in April–May (*N* = 407) and August–November (*N* = 197) of 2020 reported that 47% of SPs across clinical and management positions had been redeployed into acute or critical care at the time of the first survey, decreasing to 38% by the second survey. In both surveys, certified SPs experienced the highest rate of redeployment at 63% and 42%, respectively. Face-to-face contact with service users was reported to have been suspended by 70% of SPs (*N* = 267) in April–May and by 16% (*N* = 28) in August–November [7, 8].

The Japanese Association of Speech-Language-Hearing Therapists (JAS) conducted a public survey of 2,147 members at the peak of the first wave of COVID-19 in May 2020 [9]. The majority of respondents reported mandated use of PPE (100%), disinfection (71%), ventilation of rooms (91%), and significant organizational efforts to identify infected patients and monitor staff health (78%). Despite these changes in infection control, 27% of dysphagia assessments, which included videofluoroscopic swallow study (VFSS) and flexible endoscopic evaluation of swallowing (FEES), were being canceled. This trend in canceled services was supported by a Japanese Society of Dysphagia Rehabilitation (JSDR) survey of 112 multidisciplinary members (SPs, gastroenterologists, dentists, Ear Nose & Throat Surgeons) in August 2020 that found 67% of respondents canceled FEES or completed FEES less frequently [10].

In South Africa, existing pressures and a supply-need gap of SPs had been reported before COVID-19 [11]. Dysphagia services in this upper-middle-income country are considered more resourced than most on the continent of Africa. During the pandemic, South Africa’s ability to provide dysphagia services was compromised further with increased inequities. Practitioners reported inadequate access to PPE and reduced service provision and access to already limited instrumental assessments. Many have reported job losses or reduced income due to reductions in face-to-face client contacts and reduced access to, or engagement in, telepractice because South Africa’s SP workforce is 89% independent practitioners [11].

### Aerosol Generating Procedures

Early guidance prohibited or limited AGPs through fear of accelerating spread across healthcare workers and patients [12, 13]. SPs felt frustrated and uncertain about the accuracy of recommendations with restricted access to instrumental assessment [14]. Senior clinicians provided reassurance emphasizing the role of clinical observation and judgment in overall clinical decision-making [15]. The American Academy of Otolaryngology-Head and Neck Surgery released a statement in early May 2020 that reframed endoscopy as a procedure that is not believed to be an AGP in and of itself; rather, it may provoke AGP behaviors (e.g., coughing) [16]. After this statement, through concerns for patient care, the Centers for Disease Control and Prevention (CDC) released a statement to *avoid* delaying endoscopic procedures [17]. Many ENT and speech pathology associations restarted instrumental assessments with careful triage and enhanced PPE following this guidance [18]. ENT UK produced guidance on minimizing risk while undertaking endoscopy procedures, which the RCSLT aligned within their own guidance for resumption of therapist-led endoscopic procedures [18]. Yet, there are continuing debates about what is classified as an AGP in dysphagia management [18–23] and this lack of clarity has led to disagreements in what are appropriate levels of PPE for dysphagia assessments in some countries [1, 12]. Questions continue about the risk to staff of dysphagia interventions and appropriate infection control measures with new variants of COVID-19.

### Well-Being

In the UK, the well-being of SPs remains an increasing concern as the pandemic continues, with increased staff shortages from 7 to 20% between May and September 2020 due to sickness, self-isolation, and redeployment. Low morale and well-being issues rose from 18 to 27%, with 40% of SPs reporting anxiety [4, 6]. The sense of optimism reduced from 59 to 46% and 63% reported concern for future burnout. Similar findings were highlighted in a recent well-being survey across Ireland with 38% of the 94 SPs screened as positive for depression, 36% positive for anxiety, and 49% positive for stress [24]. At the time of submission, many countries have reinstituted non-COVID-19 elective and outpatient services to relieve the increasing waiting lists and backlogs. The continuing high numbers of patients with COVID-19 bring additional pressures to expedite hospital flow and subsequent high patient caseloads are an ongoing challenge for many SPs. Disruption to the provision of training and supervision of students and junior staff was immediate and continues in many settings. The extent of the impact of these disruptions on SPs’ well-being, training, and routine caseloads may take years to emerge.

## Dysphagia Management Innovations

The use of telehealth services have rapidly accelerated since COVID-19 [1, 25]. For some services, the uptake of telehealth has been constrained by payment and coverage issues [26] as well as infrastructure, network issues, and lack of clinical expertise [27]. Since the beginning of the pandemic and throughout 2020, Centers for Medicare & Medicaid Services (CMS) refused coverage for telehealth dysphagia services [26] only beginning coverage on March 30, 2021 [28]. In the April 2020 RCSLT survey, 61% of SPs reported conducting more telepractice via telephone than prior to COVID-19 and 44% reported conducting more telepractice via video consultation [4]. However, 69% of SPs acknowledged barriers to telehealth for up to 40% of their caseload due to lack of internet access, confidence, digital literacy, and communication impairment. Despite these challenges, interrater reliability is good to excellent, but more work is needed [29]. In South Africa, telepractice was permitted and regulated with new guidelines established by the Health Professions Council of South Africa (HPCSA) and supported by the South African Speech-Language-Hearing Association [30]. However, telehealth services were complicated with health insurance reimbursement, end-user skill level, and infrastructural barriers of limited connectivity, network access, and disruptions due to frequent electricity load shedding by the country’s electricity supplier. In response, SP students and staff from the University of KwaZulu-Natal have pioneered the “#datamustfall” movement to empower communities to build internet infrastructure (Pillay, personal correspondence). In the UK and USA, feasibility of telehealth services for rehabilitation continues to be evaluated as practical and clinical challenges remain significant.

The suspension of endoscopy procedures meant a lack of support for dysphagia management. In the UK, this led to an interest in the use of laryngeal ultrasound as a potentially safer tool. An international expert group was formed who published a rapid review of available evidence [31]. A subsequent RCSLT position statement declared that the current literature did not support ultrasound use as a stand-alone clinical swallowing assessment tool and further work would be required before it could be safely and effectively translated into clinical practice [32].

The need for PPE remains. Innovations in PPE have emerged to reduce aerosol spread and allow best practice to occur. For example, transparent masks have been produced that, in clinical trials, demonstrate improved communication and rapport building over standard face masks [33]. However, evidence of conformity to safety standards in comparison to standard masks is lacking and there is some evidence of poor acoustic performance with transparent masks which may negate their benefits for those with communication difficulties [34]. Novel transparent face respirators are also being designed and early testing confirms their safety [35]. The ‘Bubble PAPR PPE hood’ is in development by the UK National Tracheostomy Safety Project [36] and another recent early-stage innovation report describes the development of a transparent-modified full-face snorkel mask [37]. Air filtration systems and acrylic windows have been utilized to allow VFSS and FEES to proceed with reduced transmission risk and with better efficiency due to the reduced air exchange time required for rooms to be ventilated between patients [38–41]. The SNAP (Safe Nasoendoscopic Airway Procedure) device, developed by ENT surgeons in the UK, is a single-use valved endoscopic port retrofitted to any surgical mask permitting entry of a flexible endoscope, while limiting viral spread from the nasopharynx. The patient continues to wear a surgical face mask during the nasoendoscopy procedure [42], but there are limitations in its use during nasoendoscopy procedures for swallow, voice, and upper airway examinations where the surgical mask has to be lifted above the mouth to examine or feed the patient risking aerosol spread and rendering the device less useful.

## Hospitalized Patients Presenting with Dysphagia

Early in the pandemic, teams collated data on hospitalized patient cohorts describing mortality rates, rates of intensive care unit (ICU) admissions, common symptoms, and comorbidities [43–55]. A meta-analysis of 61 cohort studies with 31,089 patients found cerebrovascular disease, chronic obstructive pulmonary disease, cardiovascular disease, and malignancy among the risk factors for a poor clinical outcome in COVID-19 [50] and conditions that frequently impact swallowing physiology [56, 57]. Age has also been reported as an important risk factor with increased admission rates into ICU and mortality from COVID-19 in patients over 65 years old, especially those with comorbidities [50]. In South Africa, living with HIV and tuberculosis was independently associated with increased COVID-19 mortality [52] and featured in the clinical caseload managed by SPs. In a group of 164 COVID-19 patients referred to SP for assessment, hypertension, diabetes, and respiratory-related problems were present in 34%, 29%, and 23%, respectively [53]. One study reported that ICU patients with COVID-19 had a higher incidence of neurological disorders compared with a group of non-COVID-19 ICU patients but it is unclear whether the neurological disorders were new or pre-morbid [54].

As more patients began to recover in ICU, dysphagia became more apparent as a key focus for rehabilitation. A recent population survey of 31,129 community-dwelling adults in the USA found a 16% prevalence of reported dysphagia, suggesting that many patients with COVID-19 may also have a pre-existing dysphagia [55]. In older patients hospitalized with COVID-19, sarcopenia may also be contributing to dysphagia symptoms. As COVID-19 is primarily a respiratory illness, those with underlying respiratory conditions such as asthma and COPD are not only vulnerable to severe infection but also to an increased risk of respiratory-swallow incoordination, dysphagia, and aspiration pneumonia. Those with ongoing respiratory support, such as high-flow oxygen therapy or continuous positive airway pressure (CPAP) delivered via facemask, may struggle with eating and drinking safely, experiencing desaturation and fatigue during mealtimes. These patients need close monitoring and may benefit from compensatory strategies and diet/fluid modifications.

### Acute Care (ICU Patients)

Of the patients admitted to the hospital with COVID-19, studies report up to 20% will require ICU admission and orotracheal intubation [49, 50]. A common comorbidity of COVID-19 in critically ill patients is acute respiratory distress syndrome (ARDS), which is strongly associated with dysphagia, aspiration pneumonia, malnutrition, dehydration, and increased hospitalization [59, 60]. Moreover, it is estimated that 42%–60% of critically ill patients experience post-extubation dysphagia (PED) [61, 62], increasing risk for aspiration pneumonia, transient hypoxemia, malnutrition, extended hospitalization, and mortality [63, 64]. Pre-existing neurological disease, emergency admission, increased duration of mechanical ventilation, increased duration of renal replacement therapy, and higher severity of illness are all associated with development of post-extubation dysphagia [64]. In patients with COVID-19 who require prolonged ICU stay, such as those needing extracorporeal membrane oxygenation (ECMO), the risks for dysphagia are compounded with muscle loss and critical illness polyneuropathy [65].

**Fig. 1:**
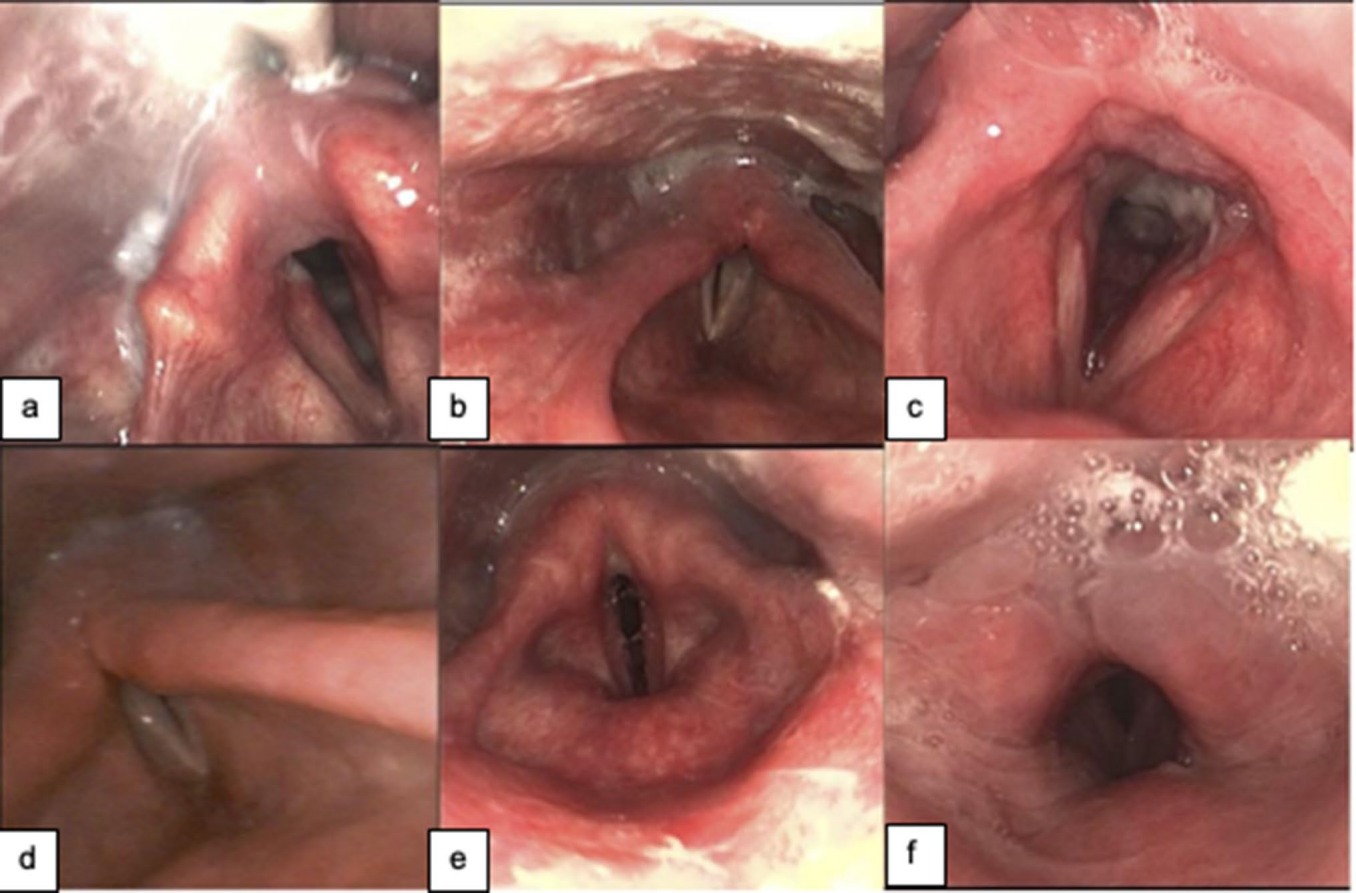
Laryngeal pathology in patients following ICU intubation. Case (a) Posterior glottic granulation tissue on right, edema and erythema of vocal folds and left arytenoid, and hyperfunction of left false vocal fold. Case (b) Inability to abduct the vocal folds due to posterior glottic stenosis. Case (c) 55-year-old male, tracheostomy & ventilated with cuff down and PMV, previously intubated for 4 weeks, 2 previous FEES kept NBM, 3rd FEES posterior subglottic granulation tissue L > R, and edematous and erythematous vocal folds with interarytenoid edema. Recommendation: safe on slightly thick fluids and minced and moist diet, effortful swallow, and PPI. Case (d) Posterior glottic stenosis and necessitated long-term tracheostomy. Case (e) 35 year old, 6 weeks in ICU, ETT 4 weeks, tracheostomy & ventilated with cuff down and PMV at time of FEES, severe erythema involving laryngeal vestibule and vocal folds, and edematous vocal folds with cystic lesions. Recommendation: safe on thin fluids and regular diet with PMV and needs PPI. Case (f) Severe diffuse laryngeal edema and excess saliva secretions. *ICU* intensive care unit, *PMV* Passy-Muir valve, *NBM* nil by mouth, *FEES* flexible endoscopic evaluation of swallowing, *L* left, *R* right, *ETT* endotracheal tube, *PPI* proton pump inhibitors

Intubation duration is an established risk factor for dysphagia [61, 63]. The longer intubation times associated with COVID-19 increase the risk of laryngeal sequelae, including the need for tracheostomy, dysphagia, vocal fold paralysis, dysphonia, edema, and laryngeal-tracheal stenosis [66, 68]. Laryngeal injury may be caused by intubation trauma, the mechanical pressure/mucosal irritation by the endotracheal tube (ETT), and disuse atrophy (Fig. 1). Patients with COVID-19 often experience prolonged intubation and delays in tracheostomy insertion [54, 69]. Literature is consistent in reporting that critical care patients with COVID-19 are intubated for longer compared with non-COVID-19 critical care patients [51, 54, 69]. Intubation duration varies between studies, perhaps due to varying sample sizes (statistical power) or acuity levels and medical practices of ICUs worldwide. One study shows 9 ± 8 days for COVID-19 patients compared with 6 ± 4 days for non-COVID-19 patients (*p* = 0.02) [54]. Other studies report average intubation durations of 24 days (*n* = 41) [69] and 15–20 days prior to tracheostomy in COVID-19 patients (*n* = 1644) [54]; *n* = 64 [51]). In the acute setting, intubation-related laryngeal injury may manifest as poor secretion management, poor cough, impaired swallowing, hoarse voice, and/or inability to protect the airway [69]. These risk factors for dysphagia in patients with COVID-19 require clinical management.

Instrumental data on dysphagia and dysphonia after COVID-19 are only just emerging because of the restrictions in the use of endoscopy and VFSS and research was paused at the outset of the pandemic (Tables 1, 2). There is early evidence of persistent sensory deficits after COVID-19. Silent aspiration is common in the general ICU population, and Lagier and colleagues found 9 of 21 patients silently aspirating on VFSS after ICU discharge for COVID-19 [70]. A prospective cohort study reported 28% (208/736) of patients admitted to their large teaching hospital with COVID-19 were referred for a swallow assessment. Of these, 102 (49%) were admitted to the ICU for mechanical ventilation and 82 (39%) received a tracheostomy [15]. Dysphagia was described as multifactorial and complicated by delirium hyperactive or hypoactive, laryngeal compromise (vocal cord palsy and or laryngeal edema), respiratory-swallow incoordination, the use of sedation, frequent expectoration of high-volume secretions, and significant fatigue [15]. Our original FEES data from the second wave reveals a high rate of laryngeal pathology in all of tracheostomized ICU patients (69 pathologies in 16 ICU patients, 14 with tracheostomy) with a median of 3 abnormalities per patient, including edema, vocal fold palsy, granulomas, and mucosal lesions (Original data [a]) (Tables 1, 2 and Fig. 1).

**Table 1:** Characteristics of dysphagia studies with published primary data from acute care.^1^

Author(s), Country, Study Type	*N* (% Male)	Age (in years)	Comorbidity/ Condition: %, Etiology	Laryngeal injuries	Hospital, ICU, Intubation, Mechanical Ventilation	Tracheostomy	Swallowing
Archer et al. [53] UK Prospective Cohort	164 (63) Hospitalized and referred to SP	Mean = 57 SD = 17	34 Hypertension 29 Diabetes 23 Respiratory 13 BMI ≥ 30 10 CHD 9 Dementia 8 CKD 8 Cancer 4 Stroke 7 Other neurological	Endoscopies completed (*n* = 11) 5 Granulomas 3 Vocal cord palsy/paresis 3 Edema	*Prevalence of Intubation* 129/164 (79%) *Intubation duration* Mean = 15 days SD = 7 days	*Tracheostomy placement* 85/164 (52%) *Time to Decannulation* *From trach insertion* Median = 19 days IQR = 16, 27 days 71% decannulated within 2 months	97% dysphagia before intervention 99 followed to hospital d/c: 31% with dysphagia
Boggiano et al. [68] UK Retrospective cohort	16 (69%) Hospitalized, referred to SP for FEES following intubation and/ or tracheostomy	Median = 56 IQR = 43–63	9 Hypertension 7 Diabetes 4 Obesity 3 Asthma 1 IHD 2 Hypercholesterolemia 2 Gout 2 Hypothyroidism 1 Cancer 4 Stroke 11 Other	Median 3 (IQR 2–4) laryngeal abnormalities; 63% clinically significant Edema 12 (75%) Abnormal movement 12 (75%) Atypical lesions 11 (69%) Erythema 6 (68%) Airway patency effecting tracheostomy weaning 8 (50%)	*Days in ICU* Median = 51 days *Intubation Duration* Median = 27 days	*Tracheostomy placement* 14 (88%) *Time to Decannulation* Median 34 days	*FEES* Signs of dysphagia 16 (100%) Aspiration 8 (50%) Silent aspiration 7 Targeted dysphagia therapy required 7 (44%)
Dawson et al. [15] UK Prospective Cohort	736 hospital *admissions* 720 (98%) admitted > 3 days 208 (29%) referred for swallowing assessment	Mean = 68 SD = 18	-	5 Vocal cord palsy Unquantified laryngeal edema Secretions with expectoration	*ICU admissions* *Study-wide* 204/720 (28%) intubated *Referred to SLP* 102/204 (50%) *Intubation duration* *Oral ETT only* Mean = 10 days SD = 6 days *Oral ETT before* *tracheostomy placement* Mean = 14 days SD = 4 days	*Tracheostomy placement* *ICU admissions* 82/204 (40%) *Referred to SLP* 82/102 (80%)	*Oral Intake Started* *From oral extubation* Mean = 5 days SD = 2 days *From trach insertion* Mean = 15 days SD = 7 days *IDDSI Level* *ICU* 2%: Level 7 33%: Levels 1–6 67%: NPO *Ward* 29%: Level 7 22%: NPO *Hospital discharge* 63%: Level 6/7 7%: NPO
Dziewas et al. [106] Germany Prospective Case Series	6 (100) Hospitalized, tracheostomized patients who survived ARDS and intubation	Median = 58 IQR = 52,60	*Comorbidity by Patient* *Patients 1, 2, 6* None *Patient 3* Hypertension, CHD *Patient 4* Hypothyroidism *Patient 5* Morbid obesity, CHF, atrial fibrillation	2 Unilateral vocal fold palsy 1 Bilateral vocal fold adductor paresis 1 irregular arytenoid cartilage movement	*Duration of Mechanical Ventilation* Median = 22 days IQR = 14, 30 days	*Tracheostomy placement* 6/6 (100%) Placement timing *from oral intubation* Median = 8 days IQR = 6, 9 *Decannulation* 3/6 (50%) *Time to Decannulation* *Post-intubation* Median = 38 days IQR = 28, 54 days	*FEES* 2 Silent aspiration 6 Reduced laryngeal sensation 3 Reduced spontaneous swallowing 3 Impaired secretion management 3 Pharyngeal weakness 1 Impaired oral control
Grilli et al. [79] Italy Prospective Case Series	41 (49%) Hospitalized	Median = 52 Range = 18–84	Exclusions: previous neurological history & sarcopenia	Not reported	None required intubation	-	*Post-acute phase of disease:* 8 had dysphagia symptoms on Volume–Viscosity Test (VVST) 2 reported swallowing difficulties on Swallowing Disturbance Questionnaire (SDQ) *6-month follow-up:* 6 / 8 resolved
Lagier et al. [70] Belgium Retrospective Cross-sectional	21 (67) Hospitalized patients who survived ARDS and intubation	Mean = 63 Range = 45–76	43 Hypertension 38 Obesity 33 Diabetes 24 OSA 29 Neurological 10 CHD		*ICU Length of Stay* Mean = 30 days *Prevalence of Intubation* 21/21 (100%) *Intubation duration* Mean = 17 days		*VFSS* Referred 0–14 days after ICU discharge 90% Dysphagia *Primary/first swallow* 6 Penetration 10 Aspiration 9 Silent *Impairments* 15 Pharyngeal delay 12 Tongue base retraction 9 Laryngeal closure 9 Oral control 7 Pharyngeal motility 5 Oral delay 3 Lip closure
Laguna et al. [108] Spain Prospective Case Series	232 (74) Admitted to ICU	Mean = 61 95%CI = 59, 62	39 Renal failure 35 Respiratory 18 Sepsis 18 Diabetes *BMI* Mean = 29 kg/m^2^ 95%CI = 28, 30		*Hospital Length of Stay* Mean = 27 days 95%CI = 26, 30 *ICU Length of Stay* Mean = 11 days 95%CI = 10, 12 *Prevalence of Intubation* 167/232 (72%) *Duration of Mechanical Ventilation* Mean = 14 days 95%CI = 11, 16 days *Prevalence of ECMO* 12/167 (7%)	*Tracheostomy placement* 67/167 (40%)	*Completed mV-VST* 93/110 (85%) survivors *Dysphagia* *Study-wide* 27/232 (12%) *Post-extubation* 25/167 (23%)
Lima et al. [55] Brazil Prospective Cohort	101 (66) Hospitalized and referred to SP	Median = 53 SD = 16	45 Hypertension 41 Pulmonary 27 Diabetes 3 Neurological		*Intubation duration* Mean = 9 days SD = 8 days		*ASHA NOMS* *24-h post-extubation* 20%: Levels 1–3 54%: Levels 4/5 *ICU discharge* 70%: Levels 6/7
Regan et al. [73] Dublin, Ireland Prospective Multi-site Cohort	100 (69) Hospitalized and referred to SP	Mean = 62 Range 17–88	21 Respiratory disease 34% Cardiology 22 Diabetes 29 Obesity	No endoscopy reported 34 GRBAS 0 51 GRBAS 1–2 14 GRBAS 3	*Prevalence of Intubation* 100% *Intubation duration* Median = 14 days IQR = 8–19.5		*Initial assessment (post-extubation)* *59: FOIS Level 1–3 (tube dependent* *31: FOIS 4–6 (modified)* *10: FOIS 7 (regular diet)* *SLT Discharge* *4: FOIS Level 1–3* *18: FOIS 4–6 (modified)* *73: FOIS 7 (regular diet)*
Wang et al. [43] China Retrospective Case Series	138 (54) Hospitalized patients	Median = 56 IQR = 42, 68	31 Hypertension 15 CHD 10 Diabetes 5 CVA 3 COPD				17% Pharyngalgia 33% In ICU

**Table 2:** Characteristics of dysphagia studies with primary unpublished data from acute care

Author(s), Country, Study Type	*N* (% Male)	Age (in years)	Comorbidity/ Condition: %, Etiology	Laryngeal injuries	Hospital, ICU, Intubation, Mechanical Ventilation	Tracheostomy	Swallowing
Pownall S et al. [d] Sheffield, UK Retrospective cohort *Unpublished data*	103 (63) Hospitalized patients referred to SP	*Mean 77* *Range 33–100*	44 Respiratory 23 Dementia 22 Deconditioned 18 Cardiovascular 11 Stroke 67% no pre-existing dysphagia		*Intubation duration* Mean = 15 days	*Duration of Tracheostomy* Mean = 25 days[MB5]	FOIS *Initial* 11% Level 1 12% Level 7 *Final* 8% Level 1 12% Level 7 29% Resolved dysphagia 17% Modified diet *Time: Assessment to Discharge* Mean = 28 days
McRae J [e] UCLH, UK Retrospective review *Unpublished data*	26 out of 77 referral to SP in ICU (73)	Mean age: 56 years Median: 57.5 Range:28–69yrs	Not recorded Nil preadmission dysphagia	Vocal cord palsy 4 Laryngeal oedema 3 Vocal cord atrophy 2 Glottic gap 2 Granuloma 1 Vocal cord nodules 1	Mean intubation time prior to trache tube: 17.2 days Median: 18 days Range: 3–33 days	*Duration of Tracheostomy* Mean: 23.3 days Median: 19 days Range: 7–53 days	*Initial Assessment* Clinical swallow assessment:100% Instrumental assessment: 42% (11/26) *Discharge outcomes:* IDDSI Level 0 and Level 7 100% Dysphonia 46% *LOS:* Mean: 47 days Median 43.5 days Range 22–116 days
Wallace S et al. [a] Wythenshawe Hospital, UK Retrospective cohort *Unpublished data*	45 (67) patients referred to SP in ICU	Median = 55 Range = 27–79 * < 60 years* 71% *60–79 years* 27% * > 80 years* 2%	27 Asthma 20 Diabetes 15 Reflux disease 15 Hypertension 15 CHD 7 High BMI 0 preadmission dysphagia		*Prevalence of Intubation* 43/45 (96%) *Intubation duration* Mean = 20.5 days Median = 18 days Range = 6–73 days	*Tracheostomy Placement* 25/45 (55%) *Duration of Tracheostomy* Mean 23 days Median = 13 days Range = 5–109 days 1 long-term	*Assessment* *Initial* 39 (87%) dysphagia FOIS—51% score 1 NBM, 36% score 2–6, 13% score normal 35 (77%) dysphonia *Final* 6 (13%) dysphagia FOIS—0 score 1 NBM, 8% score 2–6, 92% score 7 normal 12 (27%) dysphonia Initial TOMS Voice: 77% dysphonic (53% of whom scored 3 or less) Final TOMS Voice: 27% dysphonic (33% of whom scored 3 or less) Initial TOMS Swallow: 87% dysphagic (85% of whom scored 3 or less) Final TOMS Swallow: 13% dysphagic (10% score 4 mild 3% score 3 moderate
Wallace S et al. [a] Wythenshawe Hospital, UK Retrospective cohort *Unpublished data*	85 (59) patients referred to SP not in ICU	Median = 85 Range = 55–100 * < 60 years* 5% *60–79 years* 32% * > 80 years* 63%	44 Dementia 19 COPD 16 Old CVA 14 Cancer 11 Parkinson’s disease 29 preadmission dysphagia				*Assessment* *Initial* 92% dysphagia 26% NPO *Final* 77% dysphagia 4% NPO
[a] Robinson U et al. [b] Belfast H&SC Trust UK Retrospective cohort *Unpublished data*	19 (68) patients referred to SP in ICU *March–June 2020*	Median = 55 Range = 43–77	37 Cardiac 32 Diabetes 27 Respiratory 16 Neurological 5 Renal 11 None 0 preadmission dysphagia		*Prevalence of Intubation* 19/19 (100%) *Intubation duration* Median = 19 days Range = 8–52 days	*Tracheostomy Placement* 5/19 (26%) *Duration of Tracheostomy* Median = 14 days Range = 13–23 days	*Assessment* *Initial FOIS* < *7* 14/18* (78%) *Final FOIS* < *7* 8/17** (47%) *NPO/Non-oral Feedings* None *Data available for 18 out of 19 patients for initial FOIS **Data available for 17 out of 19 patients for final FOIS
Robinson U et al. [b] Belfast H&SC Trust UK Retrospective cohort *Unpublished data*	30 (80) patients referred to SP in ICU *Oct–Dec 2020*	Median = 64 Range = 42–83	30 Gastrointestinal 17 CHD 27 Respiratory 13 Diabetes 13 Renal 7 Neurological 13 None 0 preadmission dysphagia		*Prevalence of Intubation* 24/24*(100%) *Intubation duration* Median = 12 days Range = 2–42 days *data available for 24 only	*Tracheostomy Placement* 7/30 (23%) *Duration of Tracheostomy* Data available for 6/7 patients Median = 17 days Range = 8–45 days	*Assessment* *Initial FOIS* < *7* 29/30 (97%) *Final FOIS* < *7* 2/30 (7%) *NPO/Non-oral Feedings* None
Robinson U et al. [b] Belfast H&SC Trust UK Retrospective cohort *Unpublished data*	92 (54) patients referred to SP not in ICU March–June 2020	Median = 84 Range = 41–97					*Assessment* *Initial FOIS* < *7* 56/64* (88%) *Final FOIS* < *7* 34/48** (79%) *Data available for 64 out of 92 patients only **44/92 patients died during hospital stay—final FOIS rating not collected for these patients therefore data only available for 48 patients
Robinson U et al. [b] Belfast H&SC Trust UK Retrospective cohort *Unpublished data*	89 (N/A) patients referred to SP not in ICU Oct–Dec 2020	Mean = 81 Range = 60–101	17 Gastrointestinal 45 Cardiology 31 Respiratory 12 Renal 30 Dementia 45 Other Neurological 21 Diabetes 34 preadmission dysphagia				*Assessment* *Initial FOIS* < *7* 68/80* (85%) *Final FOIS* < *7* 47/61** (77%) *Data available for 80 out of 89 patients only **19/89 patients died during the hospital episode. FOIS ratings not collected for these patients therefore data available for 61 patients
Gillivan-Murphy P et al. [f] Mater Hospital Dublin Retrospective cohort *Unpublished data*	68 (51) in-patients referred to SP during hospital stay March–June 2020	Median = 75 Range = 43–97	63 Cardiology 28 COPD 25 Diabetes 25 Mental Disorder 18 Dementia 13 Intellectual Disability 1 None		*Prevalence of Intubation* 15/68 (22%) *Intubation duration* Median = 7.5 Range = 3–19	*Tracheostomy Placement* 5/68 (7%) *Duration of Tracheostomy* Median = 23 Range = 18–78	*Assessment* *FOIS* < *7* 54/64* (84%) *Data available for 64 out of 68 patients *IDDSI Liquids* ≥ *Level 1* 23/50** (46%) ** Data available for 50 out of 68 patients *IDDSI Food* < *7* 31/49*** (63%) Data available for 49 out of 68 patients

95%CI, 95% confidence interval; ASHA NOMS, American Speech-Language-Hearing Association National Outcome Measurement System; BMI, body mass index; CHD, coronary heart disease; CKD, chronic kidney disease; COPD, chronic obstructive pulmonary disease; CVA, cerebrovascular accident; EAT-10, Eating Assessment Tool-10; ECMO, extracorporeal membrane oxygenation; FOIS, Functional Oral Intake Scale; ICU, intensive care unit; IDDSI, International Dysphagia Diet Standardization Initiative; IQR, interquartile range; MV, mechanical ventilation; N/A, not available; NPO, nil per os; OSA, obstructive sleep apnea; SD, standard deviation; SLP, speech–language pathology; trach, tracheostomy tube; UK, United Kingdom; VFSS, videofluoroscopic swallow study.^1^Due to rounding, percentages may not sum to 100% in some cell

**Fig. 2:**
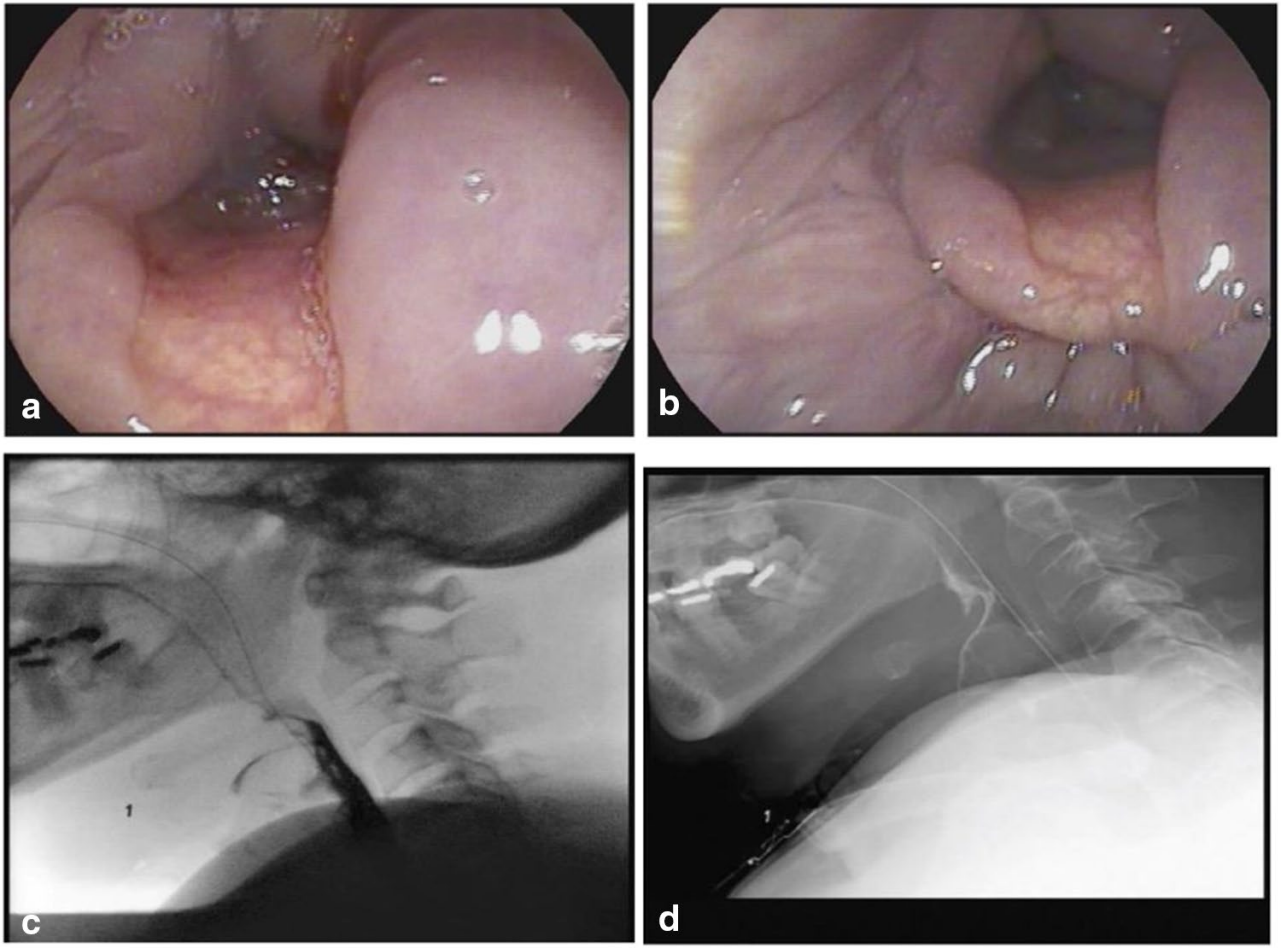
Laryngoscopy and videofluoroscopy of a 67-year-old female with COVID-19. This patient had a medical history of COPD, diabetes, OSA on CPAP at home, and previous heroin use (currently on Methadone) who was admitted to the ICU for COVID-19 progression to ARDS. She was orally intubated 3 times with a 7.5 ETT (< 90 min between intubations due to poor secretion management and stridor post-extubation), totaling 19 days and then converted to a #6 Shiley cuffed tracheostomy tube. **A** Initial view of the larynx 2 days after conversion to a tracheostomy demonstrated blood-tinged secretions flowing into the airway and a minor abrasion on the patient’s right side of the epiglottis. **B** Once secretions were cleared with suctioning, edematous tissues are readily observed in the pharynx and surrounding tissues of the larynx. **C** Initial videofluoroscopic swallow study without a one-way speaking valve due to the patient’s inability to tolerate occlusion demonstrates laryngeal penetration with a thin-liquid bolus 5 days after the endoscopy. **D** A subsequent swallow demonstrates aspiration, ultimately leading to non-oral intake and therapeutic feedings only. *COPD* chronic obstructive pulmonary disease, *OSA* obstructive sleep apnea, *CPAP* continuous positive airway pressure, *ICU* intensive care unit, *ETT* endotracheal tube, *ARDS* acute respiratory distress syndrome

Voice disorders often follow laryngeal injuries. Of our ICU patients referred to SP, 67% had a GRBAS (auditory-perceptual voice scale) ≥ 1 (*n* = 30), with 7% experiencing persistent dysphonia on discharge from hospital (Original data [b]). In another of our primary data sets, we found 77% of patients had abnormal Therapy Outcome Measure of Voice (Voice TOMS) scores [71] at initial assessment, persisting in 27% at hospital discharge (Original data [a]). Within the ICU setting, management of voice difficulties primarily involved vocal hygiene advice, offering alternative augmentative communication (ACC) strategies and ensuring referral to SP/ENT clinics for follow-up on discharge. Anecdotally, patients were often focused on other rehabilitation needs, such as improving respiratory health and overall physical function during their ICU and acute hospital stays, with dysphonia complaints becoming more prominent after hospital discharge. A summary of dysphagia and dysphonia profiles in patients with COVID-19 referred to SP from ICU is displayed in Tables 1 and 2 (case example: Fig. 2).

In Italy, prevalence of dysphagia in those requiring inpatient rehabilitation was high, with 90% of 50 patients admitted to their COVID-19 rehab center requiring a modified diet or tube feeding on admission [72]. However, most cohort studies suggest that many patients with dysphagia after COVID-19 recover functional swallowing while in hospital [15, 73], with the majority of patients referred to SP regained near normal swallow function prior to hospital discharge, regardless of intubation duration or tracheostomy status [15]. However, ICU patients with a tracheostomy were more likely to be recommended a texture-modified diet than those without (87% vs. 59%) and took longer to commence oral feeding (15 days vs. 5 days in the extubated group) [15]. Primary unpublished data from the authors support this with 71% of ICU patients (*n* = 48) nil by mouth on initial SP assessment and 92% reaching normal diet (International Dysphagia Diet Standardization Initiative (IDDSI) Functional Diet Scale 8) [74] by hospital discharge, regardless of intubation duration period (Original data [a]). Similarly, sites in the UK [75] and Ireland (Original data [b]) found 100% (26/26) and 93% (28/30) had resumed normal diet at discharge, respectively (Tables 1, 2). This may be explained by resolving post-extubation laryngeal edema, improving strength, and reducing levels of respiratory support during their ICU recovery period which may have facilitated improved laryngeal sensation and synchrony between breathing and swallowing.

Early identification and management of accumulated secretions, dysphagia, and laryngeal injury are key to successful and safe multidisciplinary tracheostomy weaning and decannulation [76]. To be optimally effective, SP assessment should be as early as possible. In the UK, the Intensive Care Society (ICS) formed a multidisciplinary Rehabilitation Collaborative in April 2020 and produced a national rehabilitation framework [77]. This guidance included a detailed SP Deep-Dive section and a new multidisciplinary screening tool highlighting the importance of early dysphagia and dysphonia rehabilitation while in ICU [78]. The screening tool, Post-ICU Presentation Screen, identifies early the need for specialist assessment and facilitates development of a rehabilitation prescription during the patient journey.

## Acute Care (Non-ICU Patients)

Dysphagia in non-intubated patients is scant at the time of writing. Yet, dysphagia teams are also seeing a group of patients managed on the acute COVID-19 hospital wards, where the maximum level of respiratory support they require is non-invasive in nature or where non-invasive respiratory support is deemed their ceiling of care. These patients are admitted directly from emergency departments to general wards. We could find only one published European paper reporting on 41 non-intubated, hospitalized patients. Eight (20%) presented with dysphagia and at 6-month follow-up and only two still self-reported swallowing difficulties [79]. To supplement this minimally published area, we explored our published primary data (Tables 1, 2) and found that these patients are older (81–85 years old in acute care vs. 55–61 years old in ICU), often with a history of pre-existing dysphagia (29%–34% in non-ICU patients vs. 0% in ICU patients) and have multiple comorbidities. As a consequence, mortality rates in this cohort were high. At Wythenshawe Hospital, 26% died or became too ill for initial assessment despite a 24-h SP response after referral. For those who were seen, only 24% had a normal Functional Oral Intake Scale (FOIS) [80] score at discharge and 42% died in hospital or immediately after discharge (Original data [a]). These data show that dysphagia persisted in this non-ICU group of patients more frequently compared to patients discharged from ICU, with ~ 75% still requiring a modified diet at discharge compared to only 13% of those discharged after ICU (Original data [a]). This trend was also seen in Belfast with 78% non-ICU compared with 7% ICU patients requiring a modified diet on discharge (Original data [b]). Palliative risk feeding approaches or diet modification and safe swallow recommendations were the primary interventions.

### Inpatient Rehabilitation

The UK government predicts that up to 45% of people after COVID-19 will require some form of low-level medical input for recovery and that 4% will require more focused, ongoing, intense rehabilitation in a facility [81]. While most dysphagia resolves in acute care, a small number of patients have more complex rehabilitation needs or a slower trajectory toward recovery. These patients require intensive dysphagia therapy programs to directly target restoration of swallowing function [75]. Primary published and unpublished data estimate the involvement of 11%–13% of patients referred to SP [53, 73] (Original data [b]). Recently published data from 11 sites across Ireland found that 37 of 100 patients referred to SP post-extubation required dysphagia rehabilitation and 20 required voice rehabilitation, with all other patients resolving with compensatory strategies [73]. Many acute hospital settings are, therefore, seeing an additional caseload of patients with long-term post-COVID-19 disability presenting with a wide range of problems due to cardio-pulmonary, musculoskeletal, neurological, and psychological/psychiatric complications of the disease, compounded in many cases by deconditioning and chronic fatigue from prolonged stays in ICU [82]. This aligns with the early evidence from China that patients with COVID-19 were presenting with neurological and respiratory after-effects leading to an increased likelihood of longer term more complex dysphagia recovery trajectories [83].

Although data are lacking detail at this time, such SP-led therapy programs often included swallowing exercises and maneuvers that could be completed independently by the patient with instruction from an SP or other team member at a distance to reduce transmission risk to staff and patients [1, 3, 6, 73, 84]. Using swallow exercises with an established evidence base in other populations has anecdotally been challenging. The authors found the execution of an effortful swallow technique and Masako (tongue hold) maneuver were more successfully implemented when compared to other techniques, such as the Mendelsohn maneuver, supraglottic swallow, super-supraglottic swallow, and Shaker head lift exercise [85]. This is presumably due to associated increased respiratory demand, cognitive load, and fatigability of patients. Evidence for the efficacy or effectiveness of such therapy programs in critically ill patients is currently not available. However, as critically ill patients with dysphagia can present with acquired weakness and disuse atrophy of the skeletal musculature of the oropharynx, swallow-strengthening exercises are physiologically reasonable and appropriate to use in this population. One case study reported successful recovery of swallowing function using pharyngeal electrical stimulation [86] and another opinion paper based on preclinical evidence, proposed that electric stimulation could improve respiratory functions, inhibit SARS-CoV-2 growth, reduce pain, boost immunity, and improve the penetration of antiviral drugs [87]. While this research holds promise, further research attention is needed.

In our experience, many factors have affected or delayed the implementation of dysphagia rehabilitation programs for patients with COVID-19 in critical care, acute care, and rehabilitation settings. Among these factors are delirium, fatigue, and weakness associated with post-intensive care syndrome (PICS), taste and smell sensory changes, poor appetite, staff unable to provide visual cues for patients when wearing PPE, redeployed staff being unfamiliar with dysphagia rehabilitation programs, lack of patient caregivers’ involvement, lack of workforce capacity, and limited access to PPE.

## Implications on Long-Term Care

The paucity of research in long-term care (LTC) is concerning considering the risks of age on COVID-19 recovery and the large population of those living in LTC facilities globally. As of September 2021, 8% of people in LTC settings have died of COVID-19 in the USA, and these statistics do not account for facilities that are not specifically for the elderly such as behavioral health residential facilities and intermediate care facilities for individuals with intellectual disabilities [88]. Globally, as soon as patients are stable in acute care, they are often discharged to their LTC setting. These patients can return home with tube feeding, on ventilators with tracheostomies, and may not have had any formal dysphagia evaluation [89]. However, we were unable to find any published papers reporting COVID-19 outcomes in swallowing and voice in patients in LTC. Many LTC facilities opted not to transport residents out of the facility for any reason other than life-saving emergency procedures to decrease the spread of COVID-19, ultimately leaving facilities with the only options of clinical swallowing evaluations or mobile FEES and VFSS services for instrumental assessments [89].

**Fig. 3:**
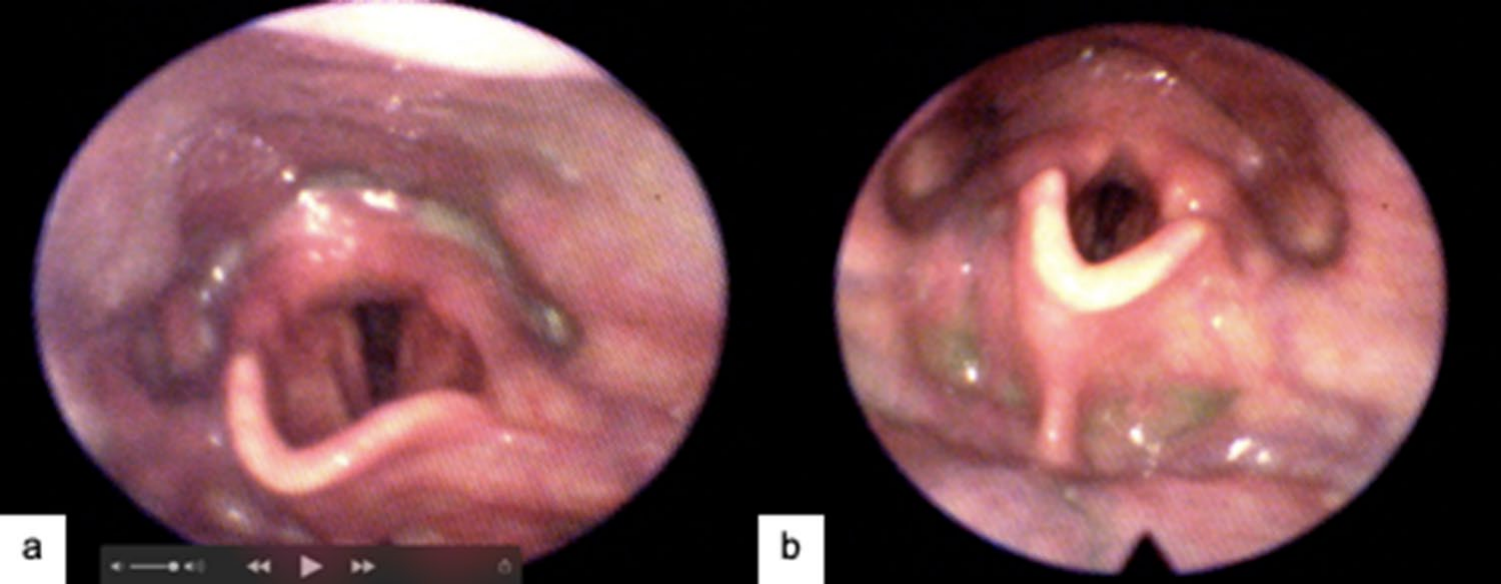
65-year old male presenting to long-term care facility post-discharge from the ICU two-week post-COVID-19 as intubated for 6 days and given a PEG tube. Past medical history: COPD and GERD. Endoscopic findings: mild dysphonia, decreased laryngeal adduction, edema, erythema, and mild sensory loss. Pt was able to be upgraded to a regular diet with thin liquids due to ability to protect the airway despite notable impairments

Anecdotal data on 47 patients following COVID-19 in a New York state LTC facility found all patients had been assessed by clinical swallowing examination in the hospital and placed on modified food and drink. FEES was completed in the skilled nursing facility on all 47 patients while they were actively infected with COVID-19 (Richard, personal correspondence). Of the 47 patients, 42 did not present with dysphagia and returned to a normal diet. The 5 patients who continued to present with difficulties swallowing were intubated during their hospital stay and presented with persisting, severe laryngeal edema. All 5 patients identified with dysphagia began dysphagia treatment (Fig. 3). Without FEES, these residents may have been left on unnecessarily modified food and drink for an extended length of time with significant risks to well-being. This reinforces the importance of swallowing assessment, with FEES in particular, in this population before hospital discharge and follow-up in LTC facilities.

## Outpatient Clinics

**Fig. 4:**
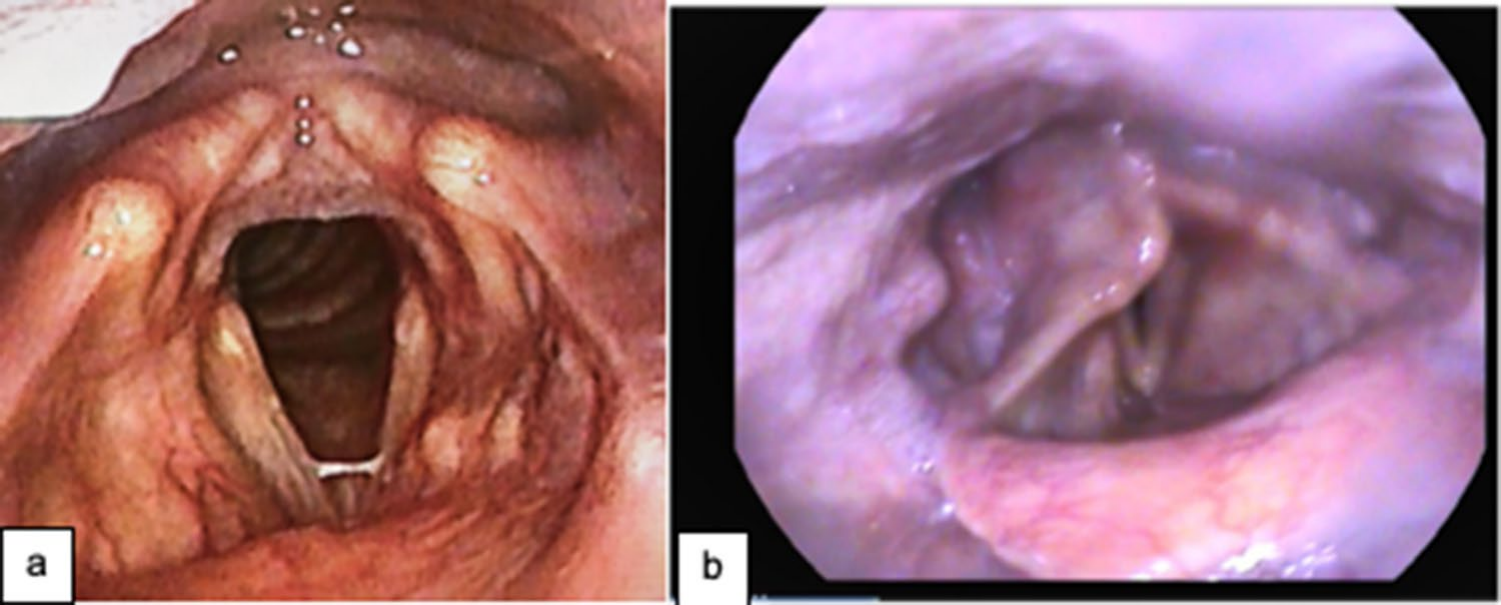
a 56-year-old male seen in outpatient clinic 7 months after hospital discharge; 28-day ventilation; and no tracheostomy. He presented with a weak voice with high-pitched quality, difficulty swallowing which he describes as a sensation of obstruction and ongoing cough. Prior to COVID-19, he was well with no medication, non-smoker, and was employed. Endoscopic findings: thinned vocal folds with complete symmetrical adduction and abduction. A possible fibrous band was visible mid-right vocal fold. **b** 58-year-old male seen in outpatient clinic six-week post-hospital discharge due to breathing/voice/swallowing issues at home. He was intubated for 33 days (size 8 ETT), had a surgically inserted size 8.0 tracheostomy, and was decannulated after 19 days (no direct laryngoscopy as an inpatient) and discharged home; was admitted from outpatient clinic for emergency airway surgery due to subglottic stenosis. Endoscopic findings: bilateral vocal fold palsy, posterior glottic stenosis, right arytenoid prolapse, and subglottic stenosis. Vocal folds in maximal abduction in this picture. Currently at home with a tracheostomy awaiting further more definitive airway surgery

**Fig. 5:**
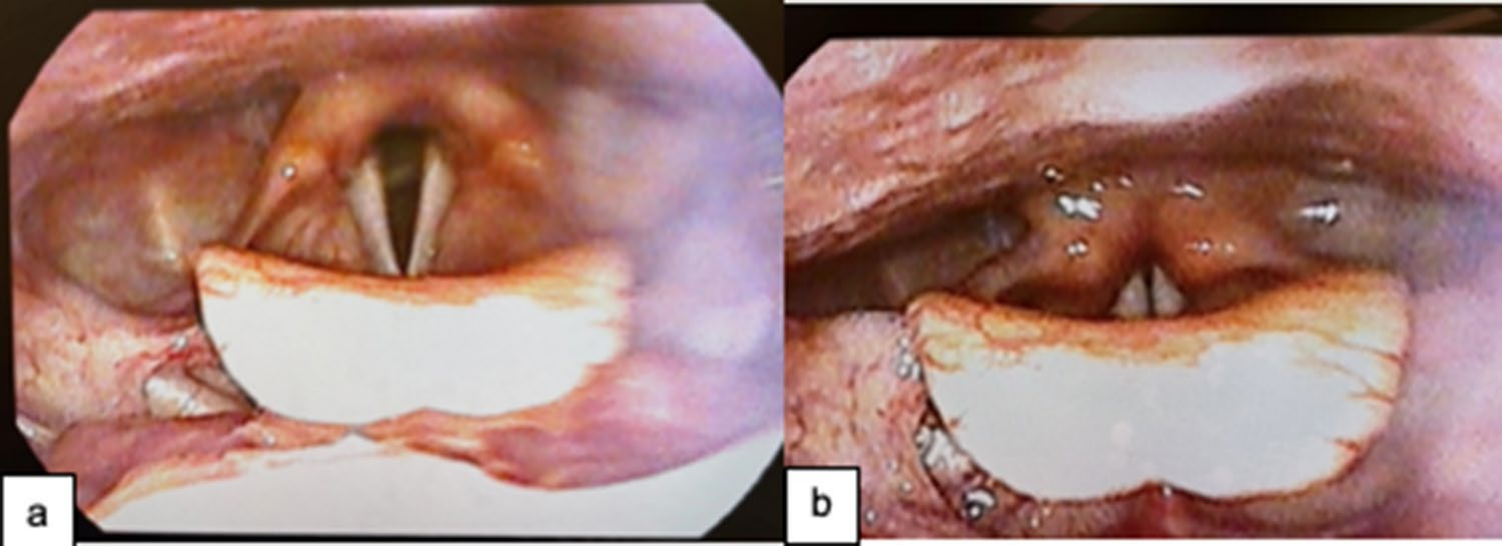
Typical laryngeal features in Long COVID-19. 49-year-old female with autoimmune deficiency and asthma was not hospitalized at time of illness. One-year post-COVID-19 still has dysphonia and ‘feeling of frog in her throat.’ Endoscopic findings: her only functional feature is significant anterior–posterior compression on vocalization

Patterns in the profiles of outpatients with persisting dysphagia and dysphonia are beginning to emerge as more patients are discharged and are followed up long term (Table 3). In one published case series, 24 patients presenting to otolaryngology with laryngeal issues following recovery from COVID-19 were described. Twenty of the patients had been hospitalized, with 18 requiring intubation. Varying laryngeal injuries, dysphonia (79%), and dysphagia (25%) were common [90]. Our original data add to this sequelae (Wallace, unpublished data; Robinson, unpublished data) with the most prevalent reported symptom dysphonia (range: 29%–79%) with a high incidence of vocal fold palsy, granuloma, arytenoid prolapse, edema, and muscle tension (Table 3; Figs. 4 and 5). Despite data from the acute setting showing functional swallowing on discharge, dysphagia is reported by 25%–58% of patients in the outpatient setting. This may indicate a disparity between managing a normal diet as indicated by an IDDSI Functional Diet Scale score = 8 [74] or FOIS = 7 [80], compared to self-reported swallowing efficiency and /or mealtime enjoyment [67]. Anecdotal reports from ICU follow-up telehealth assessments using standard triage questions indicate patients describing ongoing hypersensitivity and perceived difficulties swallowing solid foods (Wallace, personal correspondence).

**Table 3:** Patients presenting to SP outpatient clinics

Variable	Previously hospitalized patients					Non-hospitalized patients
	Naunheim et al. [109] Prospective post-discharge follow-up—recent discharge (*n* = 20)	Neeval et al. [90] Retrospective case series outpatients (*n*:24)	Rouhani et al. [69] Prospective post-discharge follow-up—average 54-day post-discharge (*n* = 41)	Ratcliffe et al. [c] Retrospective ENT outpatient follow-up (original data—unpublished) (*n* = 24)	Wallace et al. [a] Retrospective post-ICU outpatient follow-up (original data—unpublished) (*n* = 45)	Ratcliffe et al. [c] Retrospective ENT outpatient (original data—unpublished) (*n* = 21)
Sex M:F (% Male)	15:5 (75%)	12:12 50%	28:13 (70%)	16:8 (67%)	30:15 (67%)	7:14 (33%)
Age Mean (range)	59 (32–77)	50 (20–81)	56 (32–77)	56 (30–76)	55 (27–79)	48 (21–71)
Hospital journey	13 intubated (65%); 9 tracheostomy (45%)	20 (83%) hospitalized; 18 (75%) intubated	41 intubated (100%); 41 tracheostomy (100%)	24 intubated (100%); 21 tracheostomy (88%)	43 intubated (96%); 25 tracheostomy (56%)	-
Vocal fold pathologies (endoscopy, stroboscopy)	8 (40%) unilateral vocal fold immobility 3 (15%) posterior glottic stenosis 2 (10%) subglottic stenosis 2 2 (10%) granulation tissue or edema 2 (10%) LPR 2 (10%) posterior glottic diastasis 1 (5%) MTD	50% vocal fold movement impairment 39% early glottic injury 22% subglottic/ glottic stenosis 17% posterior glottic stenosis	3 (7%) unilateral vocal fold palsy 2 (4%) subglottic stenosis 1 (2%) ecchymosis right vocal fold palsy 1 (2%) bilateral vocal fold palsy	12 (50%) vocal fold palsy 6 (25%) granuloma 4 (17%) subglottic stenosis 2 (8%) arytenoid prolapse 2 (8%) oedema 2 (8%) hypofunction 1 (4%) MTD	1 (2%) glottic stenosis	10 (47%) NAD 9 (43%) MTD 2 (10%) reflux 1 (5%) vocal fold nodules 1 (5%) vocal fold pre-nodules
Breathing	7 (35%) self-reported breathing issues; 29% if not intubated	17 (70%) dyspnea 3 cough 3 respiratory distress 4 stridor	9 (22.5%) fixed upper airway obstruction on spirometry	15 (63%) self-reported breathing issues 6 (25%) chronic cough	-	17 (81%) breathing pattern disorder 11 (52%) chronic cough
Voice	12 (60%) self-reported dysphonia; 43% if not intubated	19 (79%) dysphonia 14 patient completed VRQOL: median 73 (28–100)	22/41 (53.7%) abnormal GRBAS 5/38 (13.2%) VHI: score > 11 (range 12–35)	19 (79%) dysphonia (classified by SP perceptual assessment)	13 (29%) self-reported dysphonia (telehealth by ICU outreach team, 4–6-week post-discharge home, standard triage questions)	19 (90%) dysphonia (classified by SP perceptual assessment)
Swallowing	6 (30%) self-reported dysphagia: 14% if not intubated 2 (10%) globus: 29% if not intubated 2 (10%) pain: 29% if not intubated	6 (25%) dysphagia	12/40 (30%) EAT-10 score > 2 (range 4–33) 34/41 (82.9%) FOIS 7; 3/41 (7.3%) FOIS 6; 2/41 (4.9%) FOIS 5; 2/41 (4.9%) FOIS 3	14 (58%) self-reported dysphagia 11 (46%) globus	9 (20%) self-reported dysphagia (telehealth by ICU outreach team, 4–6-week post-discharge home, standard triage questions)	3 (14%) self-reported dysphagia 16 (76%) globus

*LPR* laryngopharyngeal reflux, *MTD* muscle tension dysphonia, *FOIS* functional oral intake scale, *VHI* voice handicap index, *EAT-10* Eating Assessment Tool-10, *NAD* no abnormalities detected, *VRQOL* Voice-related quality of life Questionnaire

Breathing difficulties, ongoing chronic cough, globus, and pain continue as common symptoms after COVID-19 [90, 91] (Table 3) and are of interest to SP, particularly when considering appropriate therapeutic management. SPs who specialize in upper airway disorders may notice these symptoms with referrals as 2021 progresses. Importantly in all cohorts, new subglottic stenosis and airway problems were diagnosed at outpatient follow-up. These required surgical interventions, in some cases as an emergency (Fig. 4). SPs working with this population must be aware of patient risk for developing airway complications after discharge and have a low threshold for referral to ENT with increased work of breathing or stridor.

### Non-hospitalized Outpatient Presentations

Dysphagia and dysphonia are, however, not limited to only those hospitalized with COVID-19. There is emerging evidence that 1 in 5 people who acquired COVID-19 experience ongoing symptoms for more than 5 weeks and 1 in 10 experience symptoms for longer than 12 weeks [92]. Women are more at risk of developing ongoing symptoms [92]. Commonly described symptoms are breathlessness, cough, fatigue, cognitive impairment, and headache. Patients are also reporting a relapsing–remitting symptom profile, with new symptoms often appearing weeks after acute infection [93]. A recent survey reports symptoms in 3,762 respondents with confirmed or suspected COVID-19 across 56 countries. All respondents were more than 28-days post-first symptom and changes in voice and lump in the throat or difficulties swallowing were reported in ~ 30% [93]. Anosmia and ageusia often persist for months following acute infection and can lead to nutritional compromise [91, 94], following a similar pattern to patients after radiotherapy. Similarly, ongoing gastro-esophageal symptoms (e.g., reflux) are reported. SPs have the potential to support patients with these symptoms as part of multidisciplinary teams. This may include ensuring esophageal screening is conducted if a patient is reporting globus symptoms that may actually represent issues lower down, reinforcing body positioning while eating and resting and supporting return to trying new tastes/textures for patients with persistent ageusia.

At time of writing, it was estimated that there are 60,000 patients with ongoing rehabilitation needs following COVID-19 infection in the UK [95]. Long-COVID (defined as prolonged symptoms beyond 28 days) clinics are being implemented to provide holistic and multidisciplinary support for patients with debilitating, persisting symptoms [93]. A recent survey of patients’ experiences of long COVID found ~ 65% of respondents reported ongoing symptoms at 6 months. Most patients believed physical exercise caused a relapsing pattern, 45% required reduced workload, and 22% were not able to work at all [94].

In the UK, the National Institute for Health and Care Excellence guidance has not specifically referenced SPs as a core member of the long-COVID multidisciplinary team which may lead to variable access to SP services [96]. With increasing understanding of the condition, it is clear that SPs are receiving referrals for patients with dysphonia, chronic cough and breathing pattern disorders, and persistent dysphagia symptoms [97]. To our knowledge, there are no published papers with a primary focus on people with dysphagia or dysphonia who were not hospitalized. From our small preliminary dataset of 21 patients seen as outpatients by ENT and SP (Table 3 and Fig. 5), the presentation of non-hospitalized patients has been predominantly female (66%) with a high proportion of dysphonia (90%) and associated breathing pattern disorder (81%) as well as globus (76%), cough (52%), and dysphagia (14%) (Original data [c]). Globus-type presentations and muscle tension dysphonia and dysphagia have been reported at centers in London, UK, with one reported case of a laryngeal “tic” post-COVID-19 infection (Ratcliffe, personal correspondence). This corroborates with the 6 patients reported by Neeval et al. who had not been intubated, 4 of these with muscle tension dysphonia [90]. Outpatient ENT and voice clinics are seeing new onset idiopathic vocal fold palsies consistent with vagus nerve viral infections (Table 3). Patients with previous respiratory and laryngeal symptoms (i.e., dysphagia, dysphonia) are vulnerable to post-COVID deterioration. This potentially relates to a combination of disordered breathing and laryngeal hypersensitivity. Primary care services in the UK are reporting that 32% of patients have persistent breathlessness with 10% presenting with breathing pattern disorders [98]. SPs have the expertise to work with these patients, given training in the complex relationship between breathing, voice, and swallowing.

## The Future

Despite the current lack of studies reporting treatment efficacy for dysphagia after COVID-19, the evidence presented in this paper suggests patients will experience a myriad of physical, cognitive, and mental health rehabilitation needs [99]. A multidisciplinary approach to management is necessary [1, 64, 100]. Table 4 provides a list of ‘red flags’ for dysphagia risk that may be used to triage and support risk assessments and referral to SP. As our PPE practices in hospitals improve and become embedded in routine, we have potential to focus more fully on what we can do to rehabilitate patients instead of focusing on virus containment, ultimately creating a better understanding of the potential neurophysiological changes associated with COVID-19.

**Table 4:** Red flags for persistent dysphagia/dysphonia/laryngeal pathology

Red flags/risk factors for dysphagia		Justification/evidence
Medical history	Pre-existing dysphagia	Prevalence of pre-existing dysphagia general population is reported at 16% [55] Comorbidities of COVID-19 make likelihood of pre-existing dysphagia greater
	High BMI	Increased risk of reflux-related laryngeal injury Potential for complex and prolonged tracheostomy wean [110]
	Increased age	Higher likelihood of prolonged hospitalization and dysphagia [48] Higher likelihood of swallow decompensation, pre-morbid dysphagia, multiple comorbidities, frailty [111]
	Previous neurological disease / disorder	Pre-morbid dysphagia / dysphonia / laryngeal pathology [64]
	Chronic respiratory disease / asthma / COPD	Known relationship between COPD and silent aspiration [111] Desynchrony of respiration and swallowing [113]
Hospitalization experience	Acute Respiratory Distress Syndrome (ARDS)	Strongly associated with dysphagia, aspiration pneumonia, malnutrition [59]
	Prolonged ICU stay	Immobility/ Muscle loss/ deconditioning [65] Sepsis [114] Polyneuropathy [65] Malnutrition [115, 116] Size of ETT [61, 62]
	Prolonged intubation (incl. larger endotracheal tube > 8.0)	High risk of laryngeal injury both early and later, including paralysis, edema, stridor, and stenosis [61, 62, 96] Risk of disuse atrophy [116]
	Tracheostomy insertion	Respiratory support, laryngopharyngeal sensory impairment due to prolonged cuff inflation & lack of airflow [61, 62] Risk of secondary airway problems, for example, stenosis, vocal fold palsies, long-term tracheostomy [61, 62]
Patient complaints / concerns	Complaints of swallowing difficulties	Altered sensation, fatigue, weakness, breathlessness
	Complaints of persistent altered taste/smell & /or reflux & /or gastric issues	Increase risk of nutrition issues secondary to reduced interest in food & reduced intake
	Disturbance in voice quality following infection	High risk of laryngeal injury both early and later, including paralysis, edema, stridor, and stenosis [61, 62, 97, 116] Risk of disuse atrophy [116] Vagus nerve impairment Signification associations between severity of dysphonia, dysphagia, and cough Dysphonic COVID-19 patients are more symptomatic than non-dysphonic individuals [97]
	Ongoing fatigue on discharge	Reports of long-term fatigue for many. In those with dysphonia or dysphagia, this may have functional implications [92–94]
	Ongoing shortness of breath on discharge	Incoordination of breathing–swallowing mechanism
Occupational risk	Required to talk for prolonged periods of time with face mask Stigma Chronic fatigue, anxiety, depression	Known to lead to increase volume and increase risk of vocal pathology [117] Stigma associated with chronic cough [104, 118] High levels of anxiety & depression in long COVID [104]

Little is known about the pathophysiology of those who have lasting neurological deficits, thus treatment is currently trial and error and based on our knowledge from other populations. Much clinical research is needed for patients with persisting dysphagia to optimize outcomes. COVID-19 is primarily a respiratory disease and the long-term impact on patients with or without pre-morbid respiratory conditions who survived the infection and hospitalization is unknown. The long-term complications of COVID-19 pneumonia are still emerging, but data from previous coronavirus outbreaks such as severe acute respiratory syndrome (SARS) and Middle East respiratory syndrome (MERS) suggest that some patients may experience chronic respiratory complications, including interstitial lung disease, lung fibrosis, bronchiectasis, and pulmonary vascular disease. Along with these comes the potential for dysphagia due to uncoordinated breathing and swallowing and sequelae from COVID-19 that are only starting to be recognized. Teams should remain vigilant, monitoring for signs of dysphagia in this population while gathering data for a much-needed greater understanding. Little is known about esophageal dysphagia after COVID-19. Perhaps this lag in information is the reduction in radiology suite use in the pandemic and a preference/ ease of access to endoscopy on ICUs. It is highly likely that esophageal problems are present based on the comorbidities and medical therapies present in these patients and clinicians should consider the esophagus in their patients.

With ICU stays and intubation durations greater than the average ICU patient, it is critical to manage intubation-related laryngeal injuries and subsequent accumulated secretions, dysphagia, and dysphonia in the ICU and throughout the healthcare continuum. Dysphagia and dysphonia within the COVID-19 ICU cohort are common and often severe. Recovery is unpredictable, but many appear to resolve more rapidly and fully than in the non-ICU COVID-19 cohort. Early published data and the unpublished primary data presented in this paper suggest that this may be due to the ICU patients being younger with far fewer comorbidities than those who are not admitted to ICU. The COVID-19 ICU patients exhibit some characteristics that are uncommon of our typical non-COVID-19 ICU caseloads. Rates of laryngeal pathology and dysphonia appear to be higher, but patients have less persistent and less severe dysphagia with the exception of those who have significant neurological or respiratory deficits. The high rates of intubation trauma, laryngeal pathology, dysphonia, and dysphagia have shone a light on the value of SP intervention. Teams should capitalize on this in multidisciplinary models of care. This approach will ensure at-risk patients are identified at the earliest time post-extubation to avoid secondary complications and facilitate recovery. COVID-19 has resulted in greater awareness by the public of terminology, including ventilation, intubation, proning, and muscle weakness. It is hoped that SP staffing levels in critical care and follow-up services will increase to ensure that ongoing dysphagia and dysphonia are managed. There is a very strong argument for early identification and treatment, especially as people try to return to normal function—something only adequately staffed SP services will be able to address.

After COVID-19, SPs will have an even greater role in LTC. Patients who are more acutely ill are now being discharged to LTC, leaving SPs to manage patient acuity, tracheostomies, ventilator dependence, and PEG dependency. Patients who may have been weaned from mechanical ventilation or PEG placements will be seen in LTC settings for rehabilitation, necessitating the increased importance of access to FEES and VFSS in LTC, either via transportation to local hospitals or through the use of mobile services. COVID-19 affects many different organs other than lungs. With older populations in LTC facilities, clinicians and caregivers must remain vigilant to prevent dehydration, malnutrition, or other worsening respiratory conditions.

Being unable to work has an impact on self-esteem and financial independence [101] and being out of work is associated with poor mental health and self-harm [102]. Patients with ongoing breathing difficulties or weak voice are at risk and will need significant support from the wider healthcare team. The occupational challenges of our patients will most certainly take greater priority in 2021. Social stigma from COVID-19 for patients with chronic throat clearing, breathlessness, or stridor who are perceived as sick may be met with hostility. Anecdotally, patients have reported not being allowed to return to work in a healthcare setting as they would not ‘look’ or sound well enough to be there. Similarly, patients have found it challenging to work in hospitality around the preparation or serving of food as the perception both by the public and the employer of what meets food hygiene requirements may preclude it. Patients who now have a long-term tracheostomy may have to consider whether their old employment is viable. Additionally, the wearing of facemasks hinders intelligibility of those with dysphonia and requires increased vocal effort to be heard, impacting vocal recovery. This is a new area of potential long-term disability linked to COVID-19. We need to collate data on issues relating to vocal symptoms, vocal activity, and vocal load, to help advocate for instrumental assessment and SP support in multidisciplinary long COVID clinics. Patients will benefit from voice and breathing therapies and advice regarding amplification devices, while working collaboratively with clinicians to retrain breathing pattern disorders and strength. Patients will need support for workplace adjustments to reduce ambient noise and use of vocal rest, hydration, ration physical, and vocal commitments and prevent vocal and mental burnout during recovery.

The physical, social, and mental health impact of COVID-19 on SPs and other healthcare professionals cannot be underestimated and need to be accounted for when planning for future service delivery. Healthcare workers are at risk of contracting COVID-19 and the latest data suggest exposures are more likely from other staff in non-clinical areas such as tearooms than from patient-to-staff transmission where adequate PPE is utilized [103]. This puts pressure on staff day by day. Healthcare workers are at increased risk of burnout, exhaustion, depression, and anxiety from the intensity and relentless nature of the pandemic [104]. There are reports of healthcare workers facing stigma and being treated as pariahs due to the belief that they may transmit the virus to family members or the general public [105]. This feeling of shame has been shared anecdotally. The protracted trauma of working on the frontline, adapting ways of working, dealing with high caseloads, and the moral injury of clinical backlogs and information overload all contribute to the potential for burnout. SP service providers and managers need to ensure that measures to mitigate this are part of provision for their staff to minimize the impact.

## Conclusion

People are suffering from dysphagia and dysphonia following COVID-19 and the profiles of those hospitalized, intubated, or treated at home differ. SPs subsequently should be aware of variations in management of these disorders and expected trajectories of recovery or palliation. Those admitted to ICU often have prolonged intubations and hospital stays and, in turn, present with significant laryngeal injuries and neuropathies that may be long-lasting. Hospitalized patients may have comorbidities that either already resulted in dysphagia pre-COVID-19 or increase the risk of an acute or chronic dysphagia after a prolonged hospitalization with deconditioning. Those who present with milder illness tend to follow patterns of globus, muscle tension dysphonia, and hypersensitivity of the larynx associated with chronic cough.

The COVID-19 pandemic has highlighted health care inequities. Globally, racial and ethnic minorities, economically disadvantaged, and pregnant women have been more vulnerable [106]. This was true for high-income (minority world) countries, e.g., North America, Europe, and Australasia and for low-middle income (majority world) contexts, e.g., Africa and Asia. People with dysphagia are undoubtedly part of these overburdened, inequitable health systems. SP providers need to take steps to ensure that these groups are not overlooked. Access to vaccines is leading to international hope that the worst of the pandemic is behind us, but the huge numbers of patients who have suffered from COVID-19 will lead to pressures on healthcare providers and SPs for a long time. We need to use the burgeoning evidence base and draw on knowledge, skills, and expertise in managing dysphagia and dysphonia in other populations to maximize outcomes and advocate for patients recovering from this new virus.

## References

[1] Miles A, Connor NP, Desai R, Jadcherla S, Allen J, Brodsky M, Garand KL, Malandraki GA, McCullough TM, Moss M, Murray J, Pulia M, Riiquelme L, Langmore S (2020). Dysphagia care across the continuum: a multidisciplinary Dysphagia Research Society taskforce report of service-delivery during the COVID-19 global pandemic. Dysphagia.

[2] Miles A, Desai R, Langmore S, Connor N, Brodsky M, Malandraki G, McCullough T, Jadcherla J, Allen J, Garand K, Murray J, Riquelme L, Pulia M, Moss M. Dysphagia Research Society (DRS) Statement on COVID-19 Risk Management of Aerosol-generating Procedures (AGPs) for Dysphagia Care. Role: Chair of Dysphagia Research Society (DRS) COVID-19 Taskforce. https://www.dysphagiaresearch.org/page/COVID19AGPs (2020). Accessed 27 Mar 2021

[3] Vergara Herazo J, Skoretz SA, Brodsky MB, Miles A, Langmore S, Wallace S, Seedat J, Starmer HM, Bolton L, Clave P, Vaz Freitas S, Bogaardt H, Matsuo K, Madeira de Souza C, Figueiredo ML (2020). Assessment, diagnosis and treatment of dysphagia in patients infected with SARS-CoV-2: a review of the literature and international guidelines. Am J Speech Lang Pathol IF.

[4] RCSLT. Impact of the COVID-19 pandemic on the speech and language therapy profession. https://www.rcslt.org/learning/rcslt-guidance/#section-7 (2020). Accessed 1 Aug 2020.

[5] Chadd K, Moyse K, Enderby P (2021). Impact of COVID-19 on the speech & language therapy profession and their patients. Front Neurol.

[6] RCSLT. Ongoing impact of COVID-19 on the speech and language therapy profession - Follow Up survey. Retrieved 1st December, 2020, from https://www.rcslt.org/learning/rcslt-guidance/#section-7 (2020). Accessed 1 Dec 2020.

[7] Müller N, Kirkpatrick V, Lyons R, Antonijevic-Elliott. The impact of the Covid-19 pandemic on Speech and Language Therapists and their services in Ireland: A brief snapshot from two surveys. IASLT Newsletter, March (2021). Accessed 27 Mar 2021.

[8] Müller N, Antonijevic-Elliott S, Devlin AM, Kirkpatrick V, Lyons R. The impact of the Covid-19 pandemic on SLT services: Irish perspectives (in review). Accessed 27 Mar 2021.

[9] Yasuhiro Matsuo, Safety Department. A Report on the Urgent Survey Related to COVID-19 infection -Protecting Speech-Language Pathology Patients, Speech-Language Pathologists, and Staff-. Japanese Association of Speech-Language-Hearing Therapists Web. https://www.japanslht.or.jp/article/article_1456.html (2020). Accessed 22 Dec 2020.

[10] Medical Review Committee of Japanese Society of Dysphagia Rehabilitation. Report of “survey of the condition of Dysphagia evaluation and treatment to Covid-19 patients” Survey during the first wave of Covid-19 infection (February to June, 2020). Japanese Society of Dysphagia Rehabilitation Web. https://www.jsdr.or.jp/wp-content/uploads/file/news/news_20200923.pdf (2020). Accessed 3 Jan 2021.

[11] Pillay M, Tiwari R, Kathard H, Chikte UME (2020). Sustainable workforce—South African audiologists and speech therapists. Hum Resour Health.

[12] Bolton L, Mills C, Wallace S, Brady MC (2020). Aerosol generating procedures, dysphagia assessment and COVID-19: a rapid review. Int J Lang Commun Disord.

[13] Royal College of Speech Language Therapists (RCSLT). Interim guidance to recommence urgent and essential endoscopy in May 2020 https://www.rcslt.org/wp-content/uploads/media/docs/Covid/RCSLT-COVID-19-SLT-led-endoscopic-procedure-guidance_FINAL-(2).PDF?la=en%26hash=8101575091FE8F1ABA41B4B472387DAFB023A39D) (2020). Accessed 17 March 2021.

[14] Brodsky MB, Gilbert RJ (2020). The long-term effects of COVID-19 on dysphagia evaluation and treatment. Arch Phys Med Rehabil.

[15] Dawson C, Capewell R, Ellis S, Matthews S, Adamson S, Wood M, Sharma N (2020). Dysphagia presentation and management following coronavirus disease 2019: an acute care tertiary centre experience. J Laryngol Otol.

[16] American Academy of Otolaryngology-Head and Neck Surgery. Guidance for Return to Practice for Otolaryngology-Head and Neck Surgery [May 7, 2020]. https://www.entnet.org/sites/default/files/guidance_for_return_to_practice_part_1_final_050520.pdf. Accessed 10 Apr 2021.

[17] Centers for Disease Control and Prevention. https://www.cdc.gov/coronavirus/2019-ncov/hcp/infection-control-recommendations.html Accessed 10 Apr 2021.

[18] Royal College of Speech Language Therapists (RCSLT). Revised further in October 2020 to return to the usual FEES protocol and risk assess PPE. https://www.rcslt.org/wp-content/uploads/media/RCSLT-COVID19-SLTled-endoscopic-procedure-guidance151020.pdf (2020). Accessed 17 Mar 2021.

[19] American Speech-language-hearing Association (ASHA). Guidance to SLPs regarding aerosol generating procedures. https://www.asha.org/slp/healthcare/asha-guidance-to-slps-regarding-aerosol-generating-procedures/. Accessed 17 March 2021

[20] Wilson N, Corbett S, Tovey E (2020). Airborne transmission of covid-19. BMJ.

[21] Wilson NM, Marks GB, Eckhardt A, Clarke A, Young F, Garden FL, Stewart W, Cook TM, Tovey ER. medRxiv 2021.02.07.21251309. 10.1101/2021.02.07.21251309 10.1111/anae.15475 PMC8250912 33784793

[22] Evans MJ (2020). Avoiding COVID-19: Aerol guidelines. BMJ Yale.

[23] Freeman-Sanderson A, Ward EC, Miles A, de Pedro Netto I, Duncan S, Inamoto Y, McRae J, Pillay N, Skoretz SA, Walshe M, Brodsky MB (2021). A consensus statement for the management and rehabilitation of communication and swallowing function in the ICU: a global response to COVID-19. Arch Phys Med Rehabil.

[24] Rouse R, Regan J (2021). Psychological impact of COVID-19 on speech and language therapists working with adult dysphagia: a national survey. Int J Lang Commun Disord.

[25] Malandraki GA, Hahn Arkenberg RH, Mitchell S, Bauer Malandraki JL (2021). Telehealth for dysphagia across the life span: using Contemporary Evidence and Expertise to Guide Clinical Practice during and after COVID-19. Am J Speech Lang Pathol.

[26] ASHA Telepractice Services and Coronavirus/COVID-19. https://www.asha.org/Practice/Telepractice-Services-and-Coronavirus/. Accessed 17 Mar 2021.

[27] Aggarwal K, Patel R, Ravi R (2020). Uptake of telepractice among speech-language therapists following COVID-19 pandemic in India. Speech Lang Hear J.

[28] American Speech-Language-Hearing Association. [March 31, 2021]. https://www.asha.org/practice/reimbursement/medicare/providing-telehealth-services-under-medicare-during-the-covid-19-pandemic/. Accessed 10 Apr 2021.

[29] Borders JC, Sevitz JS, Malandraki JB, Malandraki GA, Troche MS (2021). Objective and subjective clinical swallowing outcomes via telehealth: reliability in outpatient clinical practice. Am J Speech Lang Pathol.

[30] Health Professions Council of South Africa (HPCSA). Telepractice guidelines for Health Professionals. https://www.hpcsa.co.za/?contentId=462&actionName=Home (2021). Accessed 17 Mar 2021.

[31] Allen J, Haines J, Govender R, Clunie G, Wallace S, Massey-Gaston C, Slinger C, Zaga C (2020). Utility of ultrasound in the assessment of swallowing and laryngeal function: a rapid review and critical appraisal of the literature. Int J Lang Commun Disord (IJLCD).

[32] Royal College of Speech Language Therapists (RCSLT). Use of Ultrasound for Swallowing and Upper Airway Assessment. https://www.rcslt.org/wp-content/uploads/2020/11/Revised-RCSLT-ultrasound-statement-Feb-2021.pdf (2020). Accessed 17 Mar 2021.

[33] Kratzke IM, Rosenbaum ME, Cox C, Ollila DW, Kapadia MR (2021). Effect of Clear vs standard covered masks on communication with patients during surgical clinic encounters: a randomized clinical trial. JAMA Surg.

[34] Corey RM, Jones U, Singer AC (2020). Acoustic effects of medical, cloth, and transparent face masks on speech signals. J Acoust Soc Am.

[35] Alenezi H, Cam ME, Edirisinghe M (2021). A novel reusable anti-COVID-19 transparent face respirator with optimized airflow. Bio-des Manuf.

[36] National Tracheostomy Safety Project. New disposable Bubble PAPR PPE hood developed by NTSP team. http://www.tracheostomy.org.uk/news/new-disposable-ppe-hood-developed-by-ntsp-team (2021). Accessed 17 Mar 2021.

[37] Measuria HD, Verma YV, Kerstein R, Tucker S (2021). Modified full-face snorkel mask: answer to the PPE crisis?. BMJ Innov.

[38] Plotas P, Kagkelaris K, Konstantopoulou A, Georgakopoulos C, Jelastopulu E (2020). The use of acrylic window as protective physical barrier against coronavirus infection in the context of voice disorders. Speech Lang Hear.

[39] Ku PKM, Holsinger FC, Chan JYK, Yeung ZWC, Chan BYT, Tong MCF, Starmer HM (2020). Management of dysphagia in the patient with head and neck cancer during COVID-19 pandemic: practical strategy. Head Neck.

[40] Goldman AR, Pahade JK, Langton-Frost NA, Hodges CA, Taylor AM, Bova G, Azadi JR. Adapting the modified barium swallow: Modifications to improve safety in the setting of airborne respiratory illnesses like COVID-19. Abdom Radiol (NY). 2021. 10.1007/s00261-021-03025-8 PMC7998084 33772613

[41] George A, Prince M, Coulson C (2020). Safe nasendoscopy assisted procedure in the post-COVID-19 pandemic era. Clin Otolaryngol.

[42] Zhou F, Yu T, Du R (2020). Clinical course and risk factors for mortality of adult inpatients with COVID- 19 in Wuhan, China: a retrospective cohort study. Lancet.

[43] Wang D, Hu B, Hu C (2020). Clinical characteristics of 138 hospitalized patients with 2019 novel coronavirus-infected pneumonia in Wuhan, China. JAMA.

[44] Yang W, Cao Q, Qin L (2020). Clinical characteristics and imaging manifestations of the 2019 novel coronavirus disease (COVID-19): a multi-center study in Wenzhou city, Zhejiang, China. J Infect.

[45] Tian S, Hu N, Lou J (2020). Characteristics of COVID-19 infection in Beijing. J Infect.

[46] Guan W-J, Ni Z-y, Hu Y (2020). Clinical characteristics of coronavirus disease 2019 in China. N Engl J Med.

[47] Grasselli G, Zangrillo A, Zanella A (2020). Baseline characteristics and outcomes of 1591 patients infected with SARS-CoV-2 admitted to ICUs of the Lombardy Region, Italy. Jama.

[48] Richardson S, Hirsch JS, Narasimhan M, Crawford JM, McGinn T, Davidson KW, Barnaby DP, Becker LB, Chelico JD, Cohen SL, Cookingham J, Coppa K, Diefenbach MA, Dominello AJ, Duer-Hefele J, Falzon L, Gitlin J, Hajizadeh N, Harvin TG, Hirschwerk DA, Kim EJ, Kozel ZM, Marrast LM, Mogavero JN, Osorio GA, Qiu M, Zanos TP (2020). Presenting characteristics, comorbidities, and outcomes among 5700 patients hospitalized with COVID-19 in the New York City area. JAMA.

[49] Zádori N, Váncsa S, Farkas N, Hegyi P, Erőss B, KETLAK Study Group (2020). The negative impact of comorbidities on the disease course of COVID-19. Intensive Care Med..

[50] Ahmed Y, Cao A, Thal A, Shah S, Kinkhabwala C, Liao D, Li D, Parides M, Mehta V, Ow T, Smith R, Schiff BA (2021). Tracheotomy outcomes in 64 ventilated COVID-19 patients at a high-volume center in Bronx, NY. Laryngoscope.

[51] Sanyaolu A, Okorie C, Marinkovic A (2020). Comorbidity and its Impact on Patients with COVID-19. SN Compr Clin Med.

[52] Western Cape Department of Health (WCDoH) in collaboration with the National Institute for Communicable Diseases, South Africa, Risk Factors for Coronavirus Disease 2019 (COVID-19) (2020). Death in a population cohort study from the Western Cape Province South Africa. Clin Infect Dis..

[53] Archer SK, Iezzi CM, Gilpin L (2021). Swallowing and voice outcomes in patients hospitalised with COVID-19: an observational cohort study. Arch Phys Med Rehabil.

[54] Lima MS, Sassi FC, Medeiros GC, Ritto AP, Andrade CRF (2020). Preliminary results of a clinical study to evaluate the performance and safety of swallowing in critical patients with COVID-19. Clinics (Sao Paulo).

[55] Adkins C, Takakura W, Speigel BMR, Lu M, Vera-Llonch M, Williams J, Almario CV (2020). Prevalence and characteristics of dysphagia based on a population-based survey. Clin Gastroenterol Hepatol.

[56] Altman KW, Yu GP, Schaefer SD (2010). Consequence of dysphagia in the hospitalized patient: impact on prognosis and hospital resources. Arch Otolaryngol Head Neck Surg.

[57] Roden DF, Altman KW (2013). Causes of dysphagia among different age groups: a systematic review of the literature. Otolaryngol Clin N Am.

[58] Wang X, Fang J, Zhu Y (2020). Clinical characteristics of non-critically ill patients with novel coronavirus infection (COVID-19) in a Fangcang Hospital. Clin Microbiol Infect.

[59] Mohan R, Mohapatra B (2020). Shedding light on dysphagia associated with COVID-19: the what and why. OTO Open.

[60] Brodsky MB, Huang M, Shanholtz C, Mendez-Tellez PA, Palmer JB, Colantuoni E, Needham DM (2017). Recovery from dysphagia symptoms after oral endotracheal intubation in acute respiratory distress syndrome survivors. A 5-year longitudinal study. Ann Am Thorac Soc..

[61] Brodsky MB, Levy MJ, Jedlanek E, Pandian V, Blackford B, Price C, Cole G, Hillel AT, Best SR, Akst LM (2018). Laryngeal Injury and upper airway symptoms after oral endotracheal intubation with mechanical ventilation during critical care: a systematic review. Crit Care Med.

[62] Skoretz SA, Flowers HL, Martino R (2010). The incidence of dysphagia following endotracheal intubation: a systematic review. Chest.

[63] Schefold JC, Berger D, Zürcher P, Lensch M, Perren A, Jakob SM, Parviainen I, Takala J (2017). Dysphagia in mechanically ventilated ICU patients (DYnAMICS): a prospective observational trial. Crit Care Med.

[64] Zuercher P, Moret CS, Dziewas R, Schefold JC (2019). Dysphagia in the intensive care unit: epidemiology, mechanisms, and clinical management. Crit Care.

[65] Ponfick M, Linden R, Nowak DA (2015). Dysphagia–a common, transient symptom in critical illness polyneuropathy: a fiberoptic endoscopic evaluation of swallowing study*. Crit Care Med.

[66] Vasanthan R, Sorooshian P, Sri Shanmuganathan V, Al-Hashim M (2021). Laryngotracheal stenosis following intubation and tracheostomy for COVID-19 pneumonia: a case report. J Surg Case Rep..

[67] McGrath BA, Wallace S, Goswamy J (2020). Laryngeal oedema associated with COVID-19 complicating airway management. Anaesthesia.

[68] Boggiano S, Williams T, Gill SE (2021). Multidisciplinary management of laryngeal pathology identified in patients with COVID-19 following trans-laryngeal intubation and tracheostomy. J Intens Care Soc.

[69] Rouhani MJ, Clunie G, Thong G, Lovell L, Roe J, Ashcroft M, Holroyd A, Sandhu G, Al Yaghchi CA (2020). Prospective study of voice, swallow, and airway outcomes following tracheostomy for COVID-19. Laryngoscope.

[70] Lagier A, Melotte E, Poncelet M, Remacle S, Meunier P (2021). Swallowing function after severe COVID-19: early videofluoroscopic findings. Eur Arch Otorhinolaryngol.

[71] RCSLT. RCSLT Online Outcome Tool. https://www.rcslt.org/speech-and-language-therapy/guidance-for-delivering-slt-services/outcome-measurement/outcome-tool-overview/. Accessed 12 April 2021.

[72] Brugliera L, Spina A, Castellazzi P, Cimino P, Tettamanti A, Houdayer E, Arcuri P, Alemanno F, Mortini P, Iannaccone S (2020). Rehabilitation of COVID-19 patients. J Rehabil Med.

[73] Regan J, Walshe M, Lavan S (2021). Post-extubation dysphagia and dysphonia amongst adults with COVID-19 in the Republic of Ireland: A prospective multi-site observational cohort study. Authorea..

[74] Steele CM, Namasivayam-MacDonald AM, Guida BT, Cichero JA, Duivestein J, Hanson B, Lam P, Riquelme LF (2018). Creation and initial validation of the International Dysphagia Diet Standardisation Initiative Functional Diet Scale. Arch Phys Med Rehabil.

[75] McRae J. (2020). Dysphagia and Dysphonia outcomes in Covid-19 patients with tracheostomy: Findings from a designated single site critical care hub European Society of Swallowing Disorders (ESSD e-poster, October 2020).

[76] McGrath BA, Ashby N, Birchall M, Dean P, Doherty C, Ferguson K, Gimblett J, Grocott M, Jacob T, Kerewala C, Macnaughton P, Magennis P, Moonesinghe R, Twose P, Wallace S, Higgs A (2020). Multidisciplinary guidance for safe tracheostomy care during the COVID-19 pandemic: the NHS National Patient Safety Improvement Programme (NatPatSIP). Anaesthesia.

[77] Intensive Care Society. Responding to COVID-19 and beyond: The Framework for Assessing Early Rehabilitation Needs following Treatment in Intensive Care. https://members.ics.ac.uk/ICS/ICS/GuidelinesAndStandards/Framework_for_assessing_early_rehab_needs_following_ICU.aspx (2020)

[78] Turner-Stokes L, Corner E, Siegert R, Brown C, Wallace S, Highfield J, Bear D, Aitken L, Montgomery H, Puthecheary Z (2021). The post-ICU presentation screen (PICUPS) and rehabilitation prescription (RP) for intensive care survivors Part I: development and preliminary clinimetric evaluation. J Intensive Care Soc (JICS).

[79] Grilli GM, Giancaspro R, Del Colle A, Quarato CMI, Lacedonia D, Foschino Barbaro MP, Cassano M (2021). Dysphagia in non-intubated patients affected by COVID-19 infection. Eur Arch Otorhinolaryngol..

[80] Crary MA, Mann GD, Groher ME (2005). Initial psychometric assessment of a functional oral intake scale for dysphagia in stroke patients. Arch Phys Med Rehabil.

[81] HM Government, NHS. (2021) Covid-19 hospital discharge service requirements. .https://assets.publishing.service.gov.uk/government/uploads/system/uploads/attachment_data/file/874213/COVID-19_hospital_discharge_service_requirements.pdf. Accessed 17 March 2021.

[82] British Society of Rehabilitation Medicine (BSRM) (2021) Rehabilitation in the wake of Covid-19: A phoenix from the ashes. https://www.bsrm.org.uk/downloads/covid-19bsrmissue1-published-27-4-2020.pdf. Accessed 27 March 2021.

[83] Mao L, Jin H, Wang M, Hu Y, Chen S, He Q, Chang J, Hong C, Zhou Y, Wang D, Miao X, Li Y, Bo Hu (2020). Neurologic manifestations of hospitalized patients with Coronavirus disease 2019 in Wuhan, China. JAMA Neurol.

[84] Kim SY, Kumble S, Patel B, Pruski AD, Azola A, Tatini AL, Nadendla K, Richards L, Keszler MS, Kott M, Friedman M, Friedlander T, Silver K, Hoyer EH, Celnik P, Lavezza A, Gonzalez-Fernandez M (2020). Managing the rehabilitation wave: Rehabilitation services for COVID-19 survivors. Arch Phys Med Rehabil.

[85] Leonard R, Kendall K (2018). Dysphagia assessment and treatment planning: a team approach.

[86] Traugott M, Hoepler W, Kitzberger R, Pavlata S, Seitz T, Baumgartner S, Placher-Sorko G, Pirker-Krassnig D, Ehehalt U, Grasnek A, Grieb A, Beham-Kacerovsky M, Friese E, Wenisch C, Neuhold S (2020). Successful treatment of intubation-induced severe neurogenic post-extubation dysphagia using Pharyngeal Electrical Stimulation in a COVID-19 survivor: a case report. J Med Case Rep.

[87] Allawadhi P, Khurana A, Allwadhi S (2020). Potential of electric stimulation for the management of COVID-19. Med Hypotheses.

[88] The COVID Tracking Project. The Long-Term Care COVID Tracker. https://covidtracking.com/nursing-homes-long-term-care-facilities (2021). Accessed 20 Sept 2021.

[89] Fong R, Tsai KCF, Tong MCF, Lee KYS (2020). Management of dysphagia in nursing homes during the COVID-19 pandemic: strategies and experiences. SN Compr Clin Med.

[90] Neevel AJ, Smith JD, Morrison RJ, Hogikyan ND, Kupfer RA, Stein AP (2021). Postacute COVID-19 Laryngeal injury and dysfunction. OTO Open.

[91] Carfì A, Bernabei R, Landi F, for the Gemelli Against COVID-19 (2020) Post-Acute Care Study Group. Persistent Symptoms in Patients After Acute COVID-19. JAMA 324(6):603–605. 10.1001/jama.2020.12603 10.1001/jama.2020.12603 PMC7349096 32644129

[92] Office of National Statistics. The prevalence of long COVID symptoms and COVID-19 complications. https://www.ons.gov.uk/news/statementsandletters/theprevalenceoflongcovidsymptomsandcovid19complications (2021). Accessed 17 Mar 2021.

[93] Ladds E, Rushforth A, Wieringa S (2020). Persistent symptoms after Covid-19: qualitative study of 114 “long Covid” patients and draft quality principles for services. BMC Health Serv Res.

[94] Davis HE, Assaf GS, McCorkell L, Wei H, Low RJ, Re’em Y, Redfield S, Austin JP, Akram A (2020). Characterizing Long COVID in an International Cohort: 7 Months of Symptoms and Their Impact. MedRexIv.

[95] National Institute for Health Research (NIHR). Living with COVID19—Second Review. Themed Review UK. https://evidence.nihr.ac.uk/themedreview/living-with-covid19-second-review/ (2021). Accessed 27 Mar 2021.

[96] National Institute for Health and Care Excellence (NICE). COVID-19 rapid guideline: managing the long-term effects of COVID-19. https://www.nice.org.uk/guidance/ng188 (2021). Accessed 27 Mar 2021. 33555768

[97] Lechien JR, Chiesa-Estomba CM, Cabaraux P (2020). Features of mild-to-moderate COVID-19 patients with dysphonia. J Voice.

[98] Greenhalgh T (2020). Management of post-acute covid-19 in primary care. BMJ.

[99] Needham DM, Davidson J, Cohen H, Hopkins RO, Weinert C, Wunsch H, Zawistowski C, Bemis-Dougherty A, Berney SC, Bienvenu OJ, Brady SL, Brodsky MB, Denehy L, Elliott D, Flatley C, Harabin AL, Jones C, Louis D, Meltzer W, Muldoon SR, Palmer JB, Perme C, Robinson M, Schmidt DM, Scruth E, Spill GR, Storey CP, Render M, Votto J, Harvey MA (2012). Improving long-term outcomes after discharge from intensive care unit: report from a stakeholders' conference. Crit Care Med.

[100] Brodsky MB, Pandian V, Needham DM (2020). Post-extubation dysphagia: a problem needing multidisciplinary efforts. Intens Care Med.

[101] Black C (2008). Working for a healthier tomorrow. Dame Carole Black’s review of the health of Britain’s working population.

[102] Public Health England (2019) Public Health matters: health and work. https://publichealthmatters.blog.gov.uk/2019/01/31/health-matters-health-and-work/. Accessed 27 March 2021.

[103] Gordon CL, Trubiano JA, Holmes NE, Chua KYL, Feldman J, Young G, Sherry NL, Grayson LM, Kwong JC (2021). Staff to staff transmission as a driver of healthcare worker infections with COVID-19. Infect Dis Heatlh.

[104] Dobson H, Malpas CB, Burrell AJ (2021). Burnout and psychological distress amongst Australian healthcare workers during the COVID-19 pandemic. Australas Psychiatry.

[105] Bagcchi S. (2020) Stigma during the COVID-19 pandemic. Lancet 20. https://www.thelancet.com/pdfs/journals/laninf/PIIS1473-3099(20)30498-9.pdf 10.1016/S1473-3099(20)30498-9 PMC7314449 32592670

[106] Turner-Musa J, Ajayi O, Kemp L (2020). Examining social determinants of health, stigma, and COVID-19 disparities. Healthcare.

[107] Dziewas R, Hufelschulte LM, Lepper J, Sackarnd J, Minnerup J, Teismann I, Ahring S, Claus I, Labeit B, Muhle P, Suntrup-Kruger S, Warnecke T, Padberg JS (2021). Dysphagia in patients with severe coronavirus disease 2019-potential neurologic etiologies. Crit Care Explor..

[108] Laguna MLB, Pilar M-N, Martínez de Lagrán Zurbano I, Esther Mor M, Carlos Pollan G, Constanza Dolores Viñas S, Pilar Ricart M. Dysphagia and mechanical ventilation in SARS-CoV-2 pneumonia: It’s real. Res Sq (2021). 10.21203/rs.3.rs-206615/v1

[109] Naunheim MR, Zhou AS, Puka E, Franco RA, Carroll TL, Teng SE, Mallur PS, Sing PC (2020). laryngeal complications of COVID-19. Laryngol Invest Otolaryngol.

[110] Weyh A, Davis S, Dolan J, Fattahi T, Fraker J, Salman SO (2020). Obese tracheostomy: a challenging path from surgery to decannulation. J Oral Maxillofac Surg.

[111] Azzolino D, Damanti S, Bertagnoli L, Lucchi T, Cesari M (2019). Sarcopenia and swallowing disorders in older people. Aging Clin Exp Res.

[112] Zheng Z, Wu Z, Liu N, Chen P, Hou P, Wang X, Fu Y, Liang W, Chen R (2016). Silent aspiration in patients with exacerbation of COPD. Eur Respir J.

[113] Gross RD, Atwood CW, Ross SB, Olszewski JW, Eichhorn KA (2009). The coordination of breathing and swallowing in chronic obstructivepulmonary disease. Am J Respir Crit Care Med.

[114] Zielske J, Bohne S, Brunkhorst FM, Axer H, Guntinas-Lichius O (2014). Acute and long-term dysphagia in critically ill patients with severe sepsis: results of a prospective controlled observational study. Eur Arch Otorhinolaryngol.

[115] Virgens IPA, Santana NM, Lima SCVC, Fayh APT (2020). Can COVID-19 be a risk for cachexia for patients during intensive care? Narrative review and nutritional recommendations. Br J Nutr.

[116] Krusciunas GP, Langmore SE, Gomez-Taborda S, Fink D, Levitt J, McKeehan J, McNally E, Scheel R, Rubio AC, Siner J, Vojnik R, Warner H, White D, Moss M (2020). The association between endotracheal tube size and aspiration (during FEES) in acute respiratory failure survivors. Crit Care Med.

[117] Similowski T, Moricot C, Nion N, Decavele M, Lavault S, Guerder A, Morelot-Panzini C, Serresse L (2021). Facemasks as a COVID-19 barrier: a window into the overlooked experience of chronic dyspnoea?. Lancet.

[118] McGonagle AK, Barnes-Farrell JL (2014). Chronic illness in the workplace: stigma, identity threat and strain. Stress Health.

